# Exploring the applications of hyaluronic acid‐based nanoparticles for diagnosis and treatment of bacterial infections

**DOI:** 10.1002/wnan.1799

**Published:** 2022-04-29

**Authors:** Mahir Mohammed, Nikita Devnarain, Eman Elhassan, Thirumala Govender

**Affiliations:** ^1^ Discipline of Pharmaceutical Sciences, College of Health Sciences University of KwaZulu‐Natal Durban South Africa; ^2^ Faculty of Pharmacy University of Khartoum Khartoum Sudan

**Keywords:** bacterial infection, drug conjugate, hyaluronic acid, surface modification, targeted nano‐drug delivery

## Abstract

Hyaluronic acid (HA) has become a topic of significant interest in drug delivery research due to its excellent properties, including biosafety, biodegradability, and nonimmunogenicity. Moreover, due to its ease of modification, HA can be used to prepare several HA‐based nanosystems using various approaches. These approaches involve conjugating/grafting of hydrophobic moieties, polyelectrolytes complexation with cationic polymers, or surface modification of various nanoparticles using HA. These nanoparticles are able to selectively deliver antibacterial drugs or diagnostic molecules into the site of infections. In addition, HA can bind with overexpressed cluster of differentiation 44 (CD44) receptors in macrophages and also can be degraded by a family of enzymes called hyaluronidase (HAase) to release drugs or molecules. By binding with these receptors or being degraded at the infection site by HAase, HA‐based nanoparticles allow enhanced and targeted antibacterial delivery. Herein, we present a comprehensive and up‐to‐date review that highlights various techniques of preparation of HA‐based nanoparticles that have been reported in the literature. Furthermore, we also discuss and critically analyze numerous types of HA‐based nanoparticles that have been employed in antibacterial delivery to date. This article offers a critical overview of the potential of HA‐based nanoparticles to overcome the challenges of conventional antibiotics in the treatment of bacterial infections. Moreover, this review identifies further avenues of research for developing multifunctional and biomimetic HA‐based nanoparticles for the treatment, prevention, and/or detection of pathogenic bacteria.

This article is categorized under:

Therapeutic Approaches and Drug Discovery > Nanomedicine for Infectious Disease
Nanotechnology Approaches to Biology > Nanoscale Systems in Biology
Therapeutic Approaches and Drug Discovery > Emerging Technologies

## Graphical abstract

Various approaches for the preparation of hyaluronic acid‐based nanoparticles to target bacterial infection

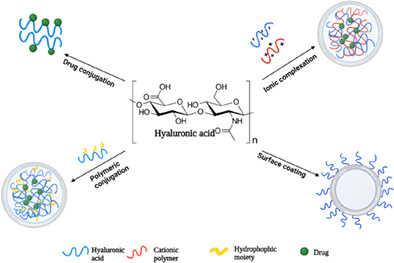

## 1 INTRODUCTION

The treatment of infectious diseases continues to be a significant priority for healthcare workers globally, as they are both a leading cause of death and have a negative impact on quality of life (Bloom & Cadarette, 2019; McArthur, 2019). Among these infectious illnesses, bacterial infections have emerged as a severe threat due to their high risk of causing premature death (Guo et al., 2020; Gupta et al., 2019; McArthur, 2019). Bacterial infections can range from mild (cutaneous) to life‐threatening illnesses, including sepsis, pneumonia, tuberculosis (TB), and others (Xiong et al., 2014). Sepsis, which is associated with bacterial infections, has significantly emerged as a bigger killer than cancer and diabetes, especially in African countries (World Health Organization, 2020b). Furthermore, the COVID‐19 research highlights that bacterial infections and its complications are more common in lethal cases than in recovered cases (Farrell et al., 2021; Shafran et al., 2021). Thus, it is essential to find effective therapy for such patients, in order to suppress bacterial infection‐related death.

Following the advent of antibiotics in 1928, it was expected that bacterial infections would be effortlessly managed (Vouga & Greub, 2016); however, practitioners have been challenged with newly emerging infectious diseases since the 1950s, posing significant health and economic concerns (McArthur, 2019). Despite their early success since their market release in 1945, conventional dosage forms of antibiotics have had many well‐documented limitations that are unrelated to antibiotic ineffectiveness and is widely acknowledged as a major contributing factor to the emergence of antimicrobial resistance (AMR). These limitations include a lack of selective targeting of the pathological site of action, poor pharmacokinetic profiles, premature drug release, insufficient tissue penetration, and rapid clearance from the blood circulation (Canaparo et al., 2019; N. Y. Lee et al., 2019). Furthermore, larger and frequent doses of antibiotics are necessary to eliminate bacterial infections, resulting in poor patient compliance and increased toxicity (A. Sharma et al., 2012). Together with widespread antibiotic misuse, these limitations have been highlighted as significant contributors to the development and spread of AMR, leading to shortening the interval between introducing antibiotics and the development of resistance (Singer et al., 2020). Multidrug‐resistant bacterial strains have rapidly exploded and become a critical worldwide problem, threatening the ability to cure infections such as TB (Singh et al., 2020), sepsis (Pant et al., 2021), and methicillin‐resistant *Staphylococcus aureus* (MRSA) infections (Nguyen & Graber, 2009), resulting in extended suffering and premature mortality. According to WHO, AMR is among the most serious international health challenges threatening humanity. Moreover, they estimated that 700,000 people would die each year globally, with the number rising to 10 million by 2050 if no fruitful efforts are made to address AMR (WHO, 2020a). For these collective reasons, discovering innovative methods that reduce toxicity to normal cells, enhance antibacterial efficacy, and slow down the progression of bacterial resistance is becoming increasingly popular in research.

Nanomedicine, which refers to the use of nanoscale materials to diagnose and treat diseases, offers a solution to address this urgent crisis (Soares et al., 2018). Nanomedicine has transformed medical technology to an advanced level, where many diseases such as cancer, AIDS, inflammation, hypertension, and bacterial infections may be treated with minor adverse effects compared to conventional dosage forms of medicines (Misra et al., 2010; Yetisgin et al., 2020). Nano‐sized drug delivery systems of antibiotics have proven to be a promising tool for overcoming the current challenges associated with current antibiotic therapy (Kalhapure et al., 2015; Muzammil et al., 2018). This could be due to several advantages of nanoantibiotics over conventional dosage forms, which include (i) selective targeting to site of infections; (ii) improved drug solubility, bioavailability, and tissue distribution; (iii) broadening the therapeutic index of drugs; (iv) sustained drug release, and (v) enhanced cellular uptake. Furthermore, nanoantibiotics enhance both pharmacokinetic and pharmacodynamic profiles of the encapsulated drug, which significantly reduces the required dose to eradicate bacterial infections (Devnarain et al., 2021; Ibrahim et al., 2021). Interestingly, in addition to improving antibacterial activity, nanoantibiotics have a substantial influence on addressing various resistance mechanisms, such as drug degradation by β‐lactamases, efflux pumps, biofilm development, and bacterial cell wall thickening (N. Y. Lee et al., 2019). However, compared to other medical diseases such as cardiovascular disease and cancer, the use of nanomedicine for the treatment of bacterial infections is still in its infancy (Misra et al., 2010; Wang et al., 2021).

To date, several types of nanodrug delivery systems, including liposomes, polymersomes, micelles, solid lipid nanoparticles (SLNs), and others, using various types of organic and inorganic materials such as polymers, lipids, or hybrid systems, have been reported in the literature for their enhanced antibacterial potential (Kalhapure et al., 2015; Muzammil et al., 2018; A. Sharma et al., 2012). Owing to their biocompatibility, ease of surface and chemical modifications, high drug loading capacity, and microenvironment responsiveness, polymer‐based NPs have gained significant attention in antibacterial applications (Arora et al., 2016; Rao et al., 2020). An interesting natural polymer that has generated increasing interest as a component of antibacterial nanodrug delivery systems over the last two decades is hyaluronic acid (HA; Arshad, Tabish, Naseem, et al., 2021; Gamarra et al., 2018; P. D. Kłodzińska, Rahanjam, et al., 2019; Özkahraman et al., 2021). Hyaluronic acid is a linear, naturally occurring mucopolysaccharide composed of alternating N‐acetyl glucosamine and d‐glucuronic acid units, which is synthesized in the plasma membrane by hyaluronan synthases (Hynes & Walton, 2000). It is also a versatile material for nanodrug delivery systems as it exists in a wide range of molecular weights starting from 6.1 kDa up to 107 kDa (Kolar et al., 2015). Additionally, it plays a crucial role in the configuration and organization of extracellular matrix, wound healing, cell adhesion control, the elasticity of connective tissue, and inflammatory modulation. Very importantly, HA is considered harmless and is rapidly degraded by hyaluronidase enzymes (HAase), making it an ideal polymer for targeted drug delivery systems (Han et al., 2015). Furthermore, the favorable characteristics of HA, such as biodegradability, nonimmunogenicity, water‐retaining activity, and selectivity toward specific receptors, have contributed to its popular use in drug delivery systems (Montanari et al., 2016). The cluster of differentiation, or CD proteins, is a group of glycoproteins that are abundant throughout the body and are considered the primary HA receptors (Dosio et al., 2016). Normally, these receptors (like CD44) are responsible for various cellular actions, including inflammation and cellular adhesion responses. In contrast, it was found to be overexpressed on several cancer cells; this phenomenon captivated the interest of researchers working on anticancer medicine. Consequently, several cancers, including ovarian, colon, breast, and squamous cancer have been extensively studied using HA nanoparticles (HA‐NPs; J. H. Kim et al., 2018; Misra et al., 2010).

Nanodrug delivery systems that consist of HA as a component have also exhibited activity against bacteria due to their intrinsic antibiofilm and bacteriostatic activity against certain bacteria (Drago et al., 2014; Pirnazar et al., 1999). Recently, CD44 receptors have been revealed to be overexpressed on human macrophages and could therefore be selectively targeted by HA‐NPs to eliminate intracellular bacterial infections (Montanari et al., 2016). Moreover, wound healing and tissue regeneration properties of HA may aid in the treatment of cutaneous infections and enable rapid recovery (Zhu et al., 2018). These exceptional abilities of HA have led to the growing interest in the formulation and development of HA‐NPs to target bacterial infections.

There have been reviews that cover in‐depth the use of HA‐based nanomaterials in the treatment of cancer, inflammatory diseases, and nucleic acids delivery (Dosio et al., 2016; J. H. Kim et al., 2018; Rao et al., 2020; Sakurai & Harashima, 2019). To the best of our knowledge, this is the first review that highlights and discusses all reported nanosystems in the literature that incorporate HA using different approaches for enhanced delivery of antibiotics to prevent, detect, and/or eradicate bacterial infections. Herein, we present an in‐depth and up‐to‐date review that highlights different techniques of preparation of HA‐NPs that have been reported in the literature. Furthermore, after an extensive analytical search of several scientific databases, we discuss and critically analyze numerous types of HA‐NPs that have been employed in antibacterial delivery to date. We have systematically categorized these HA‐NPs according to their approach for application: (i) HA‐based NPs; (ii) HA‐capped NPs; (iii) Drug‐Conjugated HA‐NPs. The benefits and limitations of these nanomaterials in the treatment of bacterial infections are thoroughly discussed and assessed. Finally, this review highlights the gaps, challenges, and future perspectives of HA‐NPs for antibacterial drug delivery.

## 2 HYALURONIC ACID AND ITS APPLICATIONS

**FIGURE 1:**
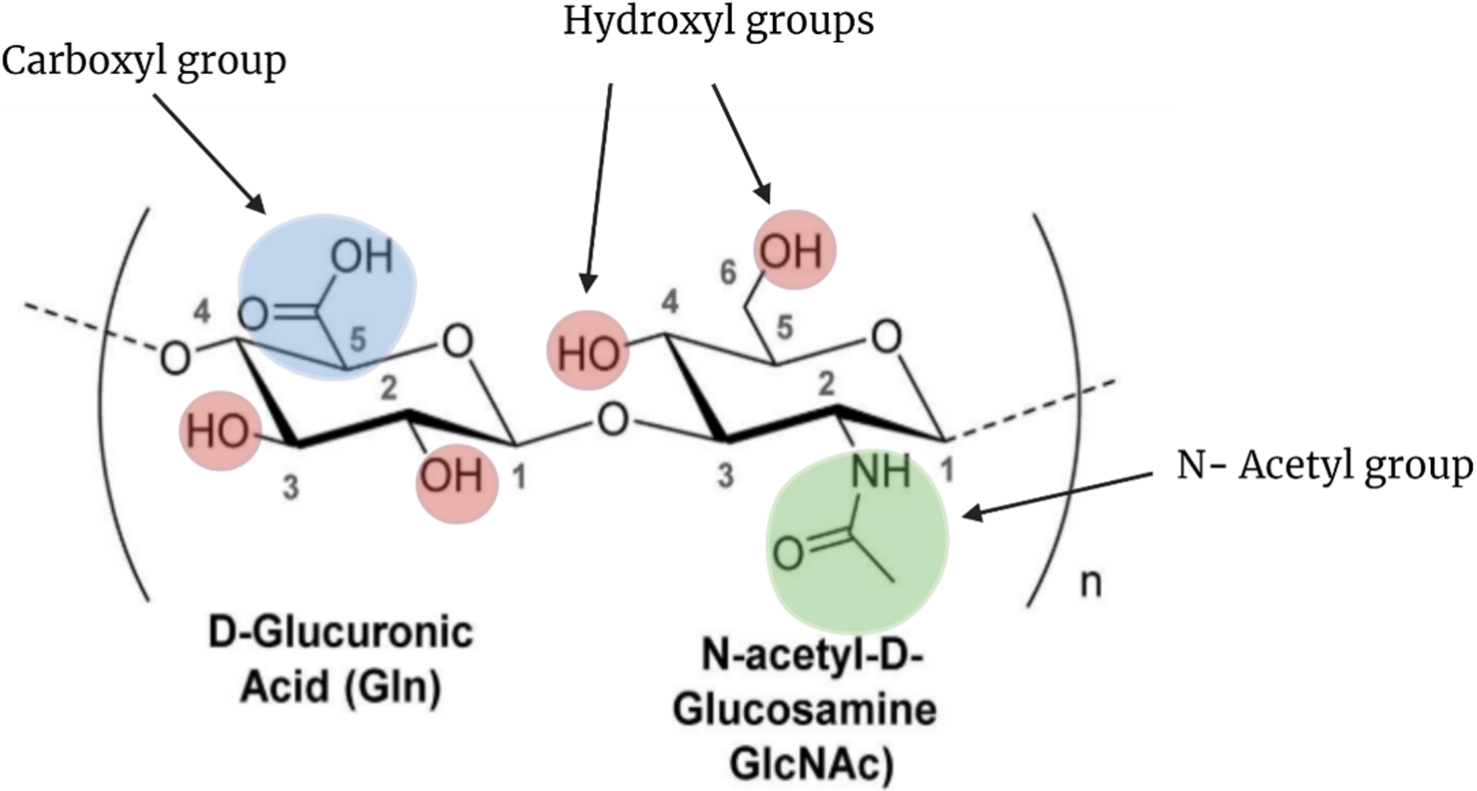
The structure of HA as well as potential locations for chemical modification of the polymer

Hyaluronic acid, conjointly termed hyaluronan, is a linear natural, polyanionic, and FDA‐approved biocompatible polymer (Figure 1), synthesized in mammals by three hyaluronan synthases (HAS1, HAS2, and HAS3; Lapčík et al., 1998). These enzymes elongate the HA backbone by repeatedly adding glucuronic acid and N‐acetyl‐d‐glucosamine groups to the growing sugar, resulting in the formation of HA with a range of molecular weights (2 × 10^5^–2 × 10^6^ Da) (Itano & Kimata, 2002). The estimated quantity of HA in the human body is 15 g, of which one‐third is replenished daily, with an average half‐life varying from 2.3 to 5.5 min. Approximately 7–8 g of this HA is found in the skin; moreover, HA is also located in connective tissue, vitreous humor, synovial fluids, and others (Necas et al., 2008). Hydrolysis of HA is predominantly by the HAase family, which may be categorized based on the producing organism into (i) mammalian HAases, which cleave the β1–4 glycosidic linkage; (ii) leech HAases, which target the β1–3 glycosidic bond; and (iii) bacterial HAase (B‐HAase), also known as hyaluronan lyase, which targets β1–4 bond like mammalian enzyme; however, the end product here is unsaturated disaccharides (Hynes & Walton, 2000). For biomedical applications, HA has been historically obtained by isolation from rooster combs; however, due to the overuse of animal rooster combs in the production of biomaterials, microbial fermentation, particularly from Streptococci species, has arisen as a new source of HA (L. Liu et al., 2011). Hyaluronic acid has attracted increased interest in the development of various drug delivery systems; however, owing to its short blood circulation time and poor enzymatic stability, several HA conjugates are prepared to overcome these limitations without affecting the value inherent characteristics of HA (Tiwari & Bahadur, 2019).

The backbone of HA consists of several carboxyl, hydroxyl, and N‐acetyl groups (Figure 1), which can be easily modified, resulting in a variety of HA derivatives. The primary approaches for modifying HA include the creation of ester and amide bonds at the carboxyl groups of the polymer in the presence of condensing agents, as well as the formation of ester and ether bonds at the hydroxyl groups of the polymer (Schanté et al., 2011). The resultant conjugates can be used to construct HA‐based delivery systems with enhanced physicochemical characteristics, including increased enzymatic stability, altered viscoelastic behavior, or controlled release (Khunmanee et al., 2017). Additionally, they are suitable for drug delivery applications by exploiting the inherent targeting characteristics of HA, minimizing immune recognition, or prolonging drug circulation (Prajapati & Maheriya, 2019).

**FIGURE 2:**
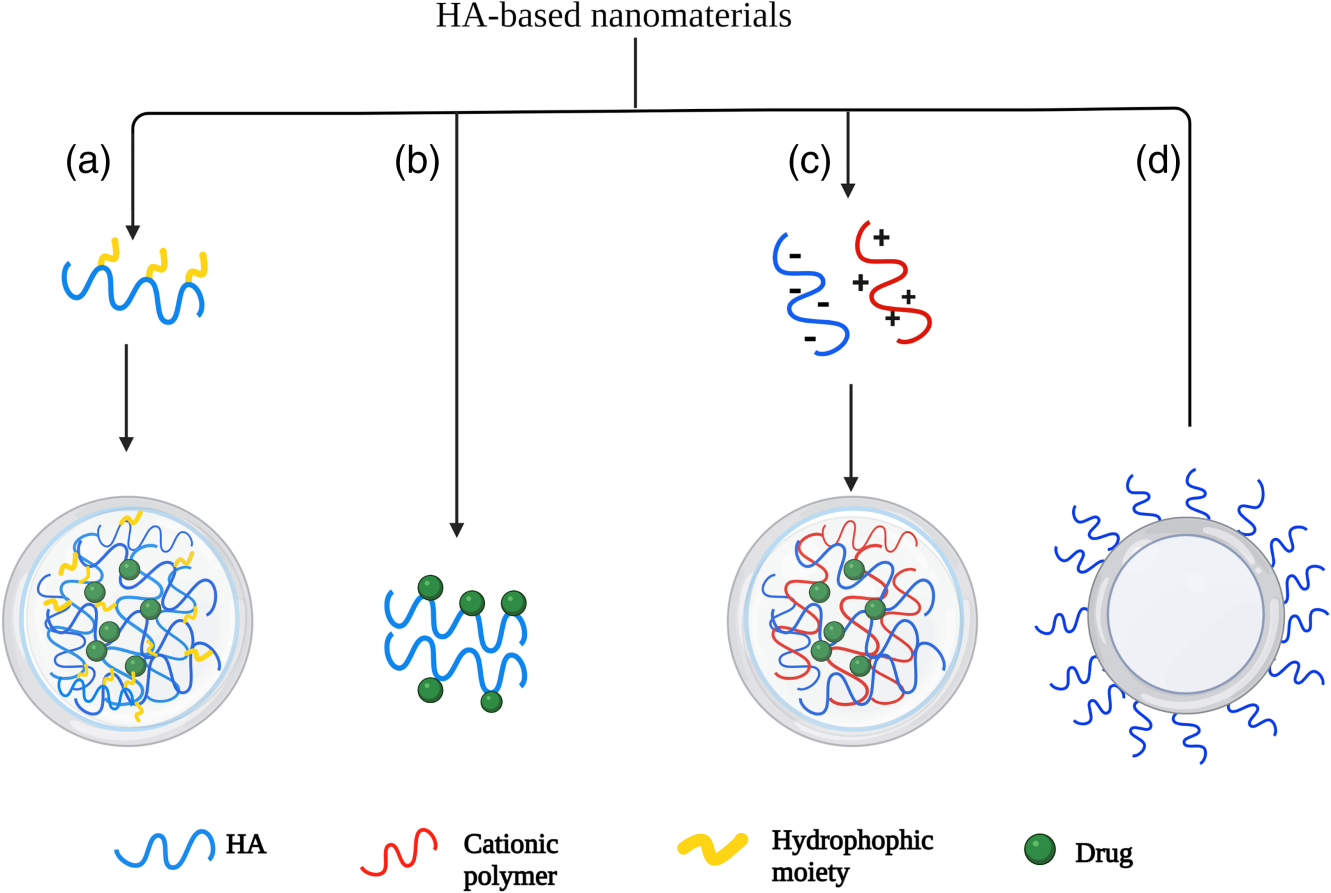
Nano‐delivery systems based on HA. Both (a) and (b) illustrate conjugation of HA with a hydrophobic moiety or drug, which self‐assemble into NPs; (c) ionic gelation of HA with cationic polymer; and (d) HA‐coated NP

**FIGURE 3:**
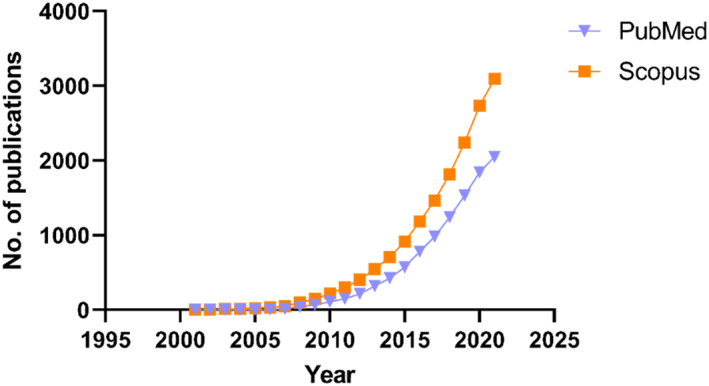
The total number of publications on HA‐based NPs. When the terms “hyaluronic acid” and “nanoparticles” were entered in PubMed (https://www.ncbi.nlm.nih.gov/pubmed/) and Scopus (https://www.scopus.com/search), the number of publications that appeared year after year was graphed

Hyaluronic acid and its conjugates have been used to formulate several types of nanocarriers, which can be prepared using three main strategies (J. H. Kim et al., 2018; Sakurai & Harashima, 2019), as shown in Figure 2. The first strategy involves conjugating/crosslinking hydrophobic moieties (including drugs) to the HA backbone, which results in the formation of amphiphilic derivatives that can be employed to formulate various types of NPs, including nanogels (Silva et al., 2016), polymersomes (Walvekar et al., 2019), micelles (Gao et al., 2014), and others (J. H. Kim et al., 2018). Owing to the anionic nature of HA, the second strategy is via ionotropic gelation with cationic polymers; this method accounts for approximately 30% of formulated HA‐based NPs (Sakurai & Harashima, 2019). Finally, the third strategy involves surface modification of various nano‐delivery systems, which is achieved by depositing an auxiliary layer(s) on the surface of the nanocarriers, which alters the properties of the nanocarrier with or without the formation of covalent bonds (K. Kim et al., 2019). The produced HA‐based NPs exhibit several desirable features, including nonimmunogenicity, biosafety, and anti‐inflammatory activity (Choi et al., 2012). HA‐based NPs have been thoroughly investigated in the field of drug delivery to enhance the biocompatibility of the material and facilitate drug delivery via passive and active targeting. Recently, the number of published articles on “hyaluronic acid” and “nanoparticles” have grown remarkably (Figure 3) (Sakurai & Harashima, 2019); interestingly, the number of published articles has reached 2019 and 3095 in PubMed and Scopus, respectively, as of the date of this review. However, compared to anticancer delivery, the application of HA in the nano delivery of antibiotics is still in its infancy.

The application of HA to develop nanomaterials for antibacterial delivery may demonstrate enhanced and synergistic activity owing to its intrinsic bacteriostatic and antibiofilm properties against certain bacterial strains (Drago et al., 2014; Pirnazar et al., 1999). Hyaluronic acid is also known to have wound healing, tissue regeneration, and anti‐inflammatory characteristics, which may aid in treating cutaneous infections and promoting rapid recovery (Zhu et al., 2018). Furthermore, numerous human macrophages have been shown to overexpress CD44 receptors, suggesting that HA‐NPs might be used to target and destroy intracellular bacterial infections selectively (Almalik et al., 2013). It has been reported that polycarboxylic acids like HA are found to reduce the pH of infection sites, thereby generating an environment where bacteria find it hard to survive (Walvekar et al., 2019). Additionally, the concept of developing HAase‐responsive antibiotic‐loaded NPs has been employed to enable on‐demand release of the encapsulated antibiotics, resulting in safe and effective antibiotic delivery (Baier et al., 2013). All these benefits make HA‐based nanomaterials an excellent candidate for eradicating both extracellular and intracellular bacteria.

The following sections review in detail HA‐based nanomaterials that have been used for antibacterial drug delivery. Herein, we systematically classified these nanomaterials into three broad groups based on the rationale behind the use of HA: (i) HA‐based nanocarriers that are conjugated, crosslinked, or ionically complexed with other molecules (fatty acids, polymers, etc.); (ii) HA‐coated NPs, where HA is utilized to modify the surface of NPs; and (iii) HA‐drug conjugate, which contains a variety of antibiotics covalently bonded to HA.

## 3 HYALURONIC ACID‐BASED NANOCARRIERS

Hyaluronic acid and its conjugates have been used to formulate various types of nanocarriers. These nanocarriers exhibited enhanced biological activity, including anticancer (Han et al., 2015), anti‐inflammatory (Rao et al., 2020), and most importantly, antimicrobial activity (Ahire & Dicks, 2016). As it is an ideal biopolymer, HA has been widely used to develop HA‐NPs as targeted antibacterial nano delivery systems (Kłodzińska et al., 2018; Montanari et al., 2018, 2020; Walvekar et al., 2019). Therefore, this section will highlight and critically analyze all previously reported HA‐based nanocarriers used for antibacterial delivery to date.

### 3.1 Nanogels

Nanogels (NGs) are spherical nanosized networks produced when polymers are chemically or physically crosslinked. They exhibit the distinct properties of both hydrogels and NPs. Nanogels can be prepared using a wide variety of polymers. At the top of the list is HA, owing to its simple conjugation methods, excellent mechanical properties, and biosafety (H. Zhang, Zhai, et al., 2016). The review on NGs shows that various HA‐NGs have been formulated with as well as without drug loading to target specific objectives (P. D. Kłodzińska, Rahanjam, et al., 2019; Montanari et al., 2020).

**FIGURE 4:**
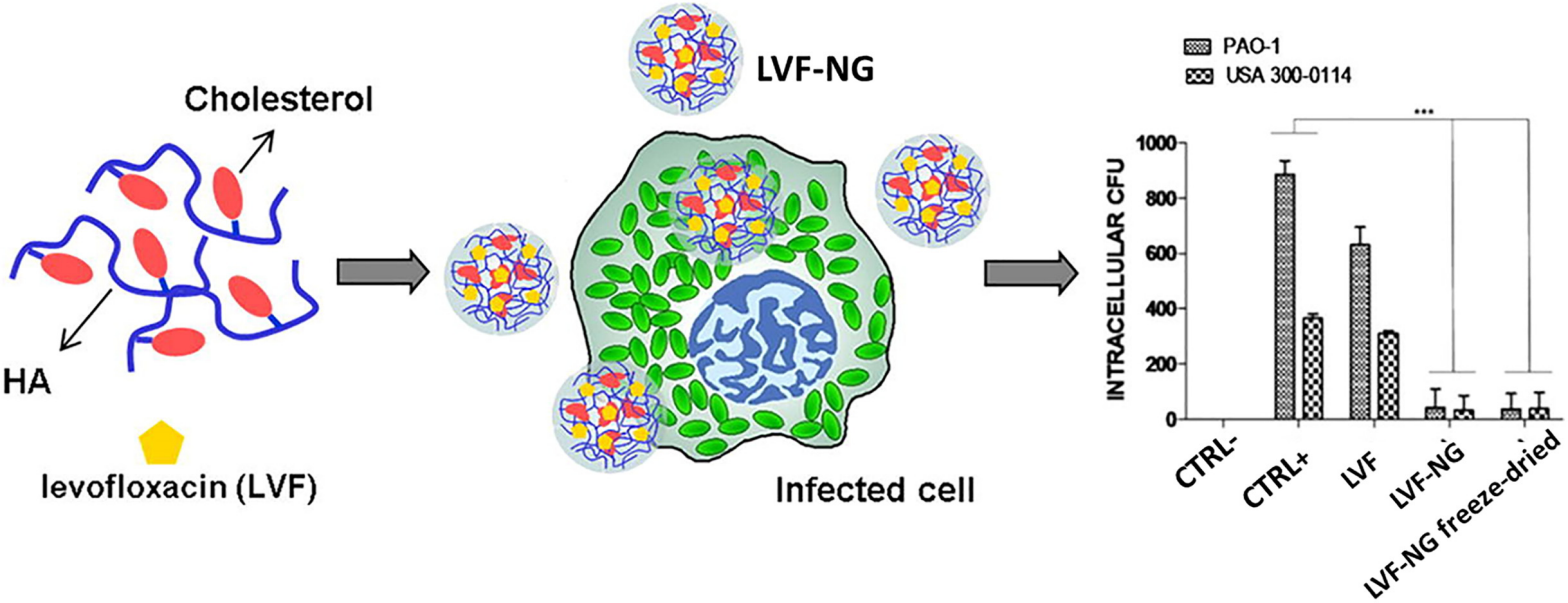
Schematic illustration of LVF‐NGs for targeting intracellular infections (Montanari et al., 2014)

**FIGURE 5:**
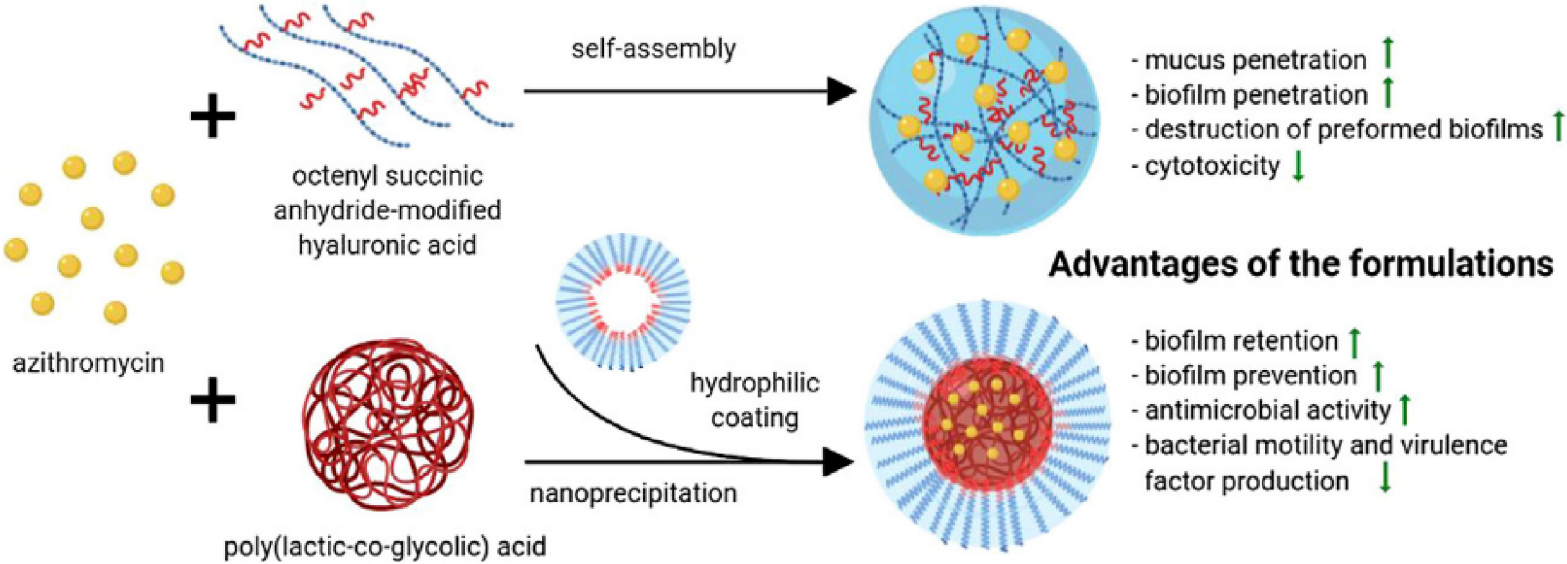
Head‐to‐head comparison of HA‐NGs and coated poly (lactic‐co‐glycolic acid) NPs for AZ delivery (Kłodzińska, Rahanjam, et al., 2019)

Various antibiotics have been encapsulated into HA‐based NGs (HA‐NGs) to eliminate bacterial infections (S. N. Kłodzińska, Wan, et al., 2019; Yuda Liu et al., 2021; Montanari et al., 2014, 2018). In this regard, Montanari et al. reported two studies on HA‐NGs encapsulated with levofloxacin (LVF) and gentamycin (GM). The HA‐NGs in these studies were prepared using the nanoprecipitation technique of cholesterol‐conjugated HA. In the first study, Montanari et al. (2014) formulated LVF‐loaded self‐assembled HA‐NGs (LVF‐NGs) targeting intracellular bacteria (Figure 4). Levofloxacin was loaded into the NGs with a drug loading efficiency of 5% and average size of 155 nm. The antibacterial studies assessed both extracellular and intracellular activity against *Staphylococcus aureus*, MRSA, and *Pseudomonas aeruginosa*. The extracellular in vitro results showed no improvement in the minimum inhibitory concentration (MIC) values of LVF‐NGs compared with the free LVF. While the intracellular studies on the human ovarian cancer cell line (HeLa) showed that LVF‐NGs significantly killed intracellular bacteria as compared to free LVF (which exhibited no activity). Interestingly, this study represents the first‐ever report of antibiotic‐loaded self‐assembled NGs (Montanari et al., 2014). In their second study, they explored the use of HA‐NGs to specifically target infected human keratinocytes. The NGs were preloaded with either GM or LVF. The mean diameters of GM‐loaded HA‐NGs (GM‐NGs) and LVF‐NGs were 250 and 350 nm, respectively, with a drug loading efficiency of 40% and 11.4%, respectively. Moreover, the antibacterial studies showed that both formulations had the same MIC and minimum bactericidal concentration (MBC) values as free antibiotics against extracellular *S. aureus*. However, intracellularly, NGs significantly enhanced the antibacterial activity of LVF, while GM and GM‐NGs showed comparable activity. This was explained by the ability of HA‐NGs to shift the intracellular fate of LVF from the cytosol to the lysosomes, therefore enhancing their activity. In contrast, GM, an antibiotic mainly accumulates in lysosomes, exhibits considerable intracellular activity even without loading into NGs (Montanari et al., 2018). Although the drug loading efficiency in the first study was lower than in the second, both studies demonstrated a substantial improvement in intracellular antibacterial activity with no change in extracellular activity. Furthermore, unlike their former study, this study investigated biosafety and drug release studies, which are critical in nanoantibiotics characterization. Further in vivo studies are required in order to provide a step forward toward reaching the clinical stage. Another antibiotic azithromycin (AZ) was explored in a study by P. D. Kłodzińska, Rahanjam, et al. (2019) where they evaluated and compared HA‐NGs and coated poly(lactic‐co‐glycolic acid) NPs (T‐PLGA NPs) in a head‐to‐head comparison to determine the relative benefits and drawbacks of each as promising delivery system (Figure 5). The microfluidic technique was used to prepare the HA‐NGs, followed by AZ loading. Compared to T‐PLGA NPs, AZ‐loaded NGs were larger in size and had a higher EE %; nevertheless, both had a diameter of less than 200 nm. The in vitro cytotoxicity assay revealed that loaded HA‐NGs are relatively nontoxic to HepG2 and lung epithelial cells (Calu‐3), but T‐PLGA NPs exhibit some toxicity. Although both delivery systems enhanced the antibacterial and anti‐virulence activity of AZ, AZ‐loaded NGs enhanced the penetration of AZ into biofilms and also eliminated preformed biofilms more effectively than T‐PLGA NPs (S. N. Kłodzińska, Wan, et al., 2019). Nevertheless, this study lacked in vitro drug release and in vivo antibacterial studies which may add further biological validation to such promising nanosystem. A recent study by Yuda Liu et al. (2021) further expanded the performance of HA‐NGs in antibacterial delivery. They reported novel targeted “on‐demand” delivery of composite nanosystems based on the triple controlled release of inclusion composites (IC), polymeric NPs, and HA‐NGs. The ultimate objective of this investigation was to efficiently eliminate *S. aureus*. In this regard, enrofloxacin was incorporated into IC and then dispersed into poloxamer 188 coating NGs formulated by ionic complexation between chitosan (CS) and HA. Nanosystems with mean EE%, sizes, and PDI of 95.4%, 118.8 nm, and 0.26, respectively, were prepared. By incorporating IC into the HA‐NGs, the nanosystem gained multifunctional characteristics by releasing enrofloxacin in a dual pH/HAase‐responsive manner at the infection site, therefore reducing premature release of the drug. Furthermore, the composite nanosystems were able to be absorbed onto the surface of *S. aureus*, resulting in increased antibacterial activity (Liu et al., 2021). Unlike the above HA‐NGs, this is the first stimuli‐responsive HA‐NGs encapsulated with an antibacterial drug. This study established that by cleverly integrating IC with HA‐NGs stabilized with poloxamer 188, the developed composite nanosystems may reduce premature drug release and improve targeting to *S. aureus*. Therefore, this study may serve as a fruitful approach to tackle the treatment difficulties associated with *S. aureus* as well as other bacterial infections. In conclusion, the studies described in this paragraph could lay the ground for expanding the use of HA‐NGs to enhance antibiotics delivery due to their ability to enhance intracellular antibacterial activity, alter the fate of several antibiotics, provide on‐site antibiotic release, and, most importantly, eliminate side effects associated with loaded antibiotics.

**FIGURE 6:**
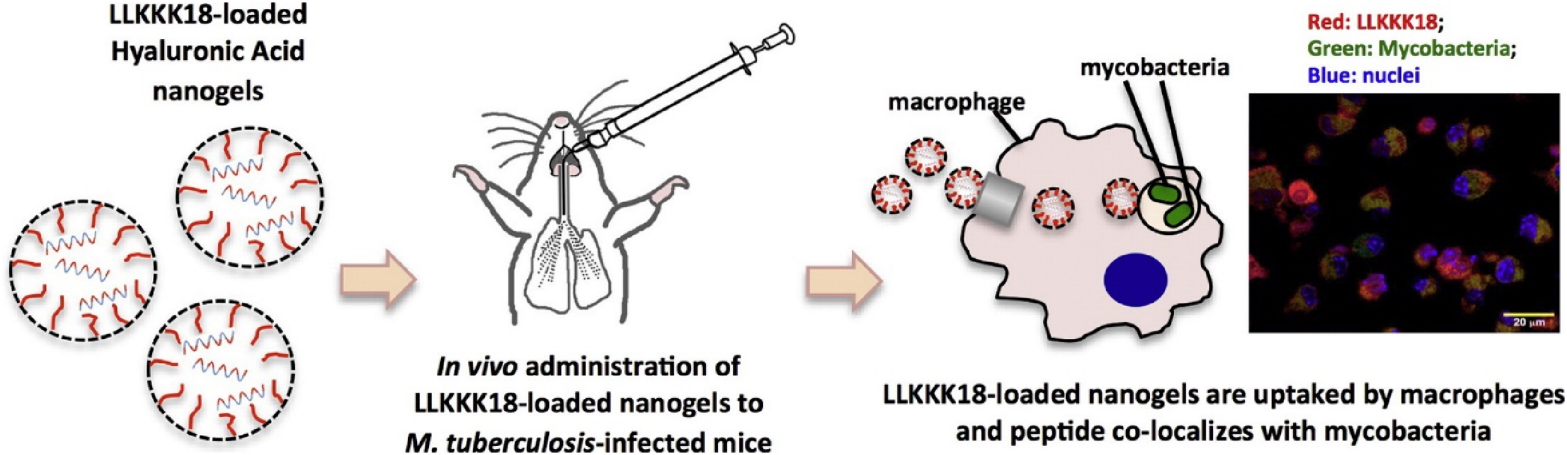
Schematic illustration of LLKKK18‐loaded HA‐NGs targeting intracellular mycobacteria (Silva et al., 2016)

**FIGURE 7:**
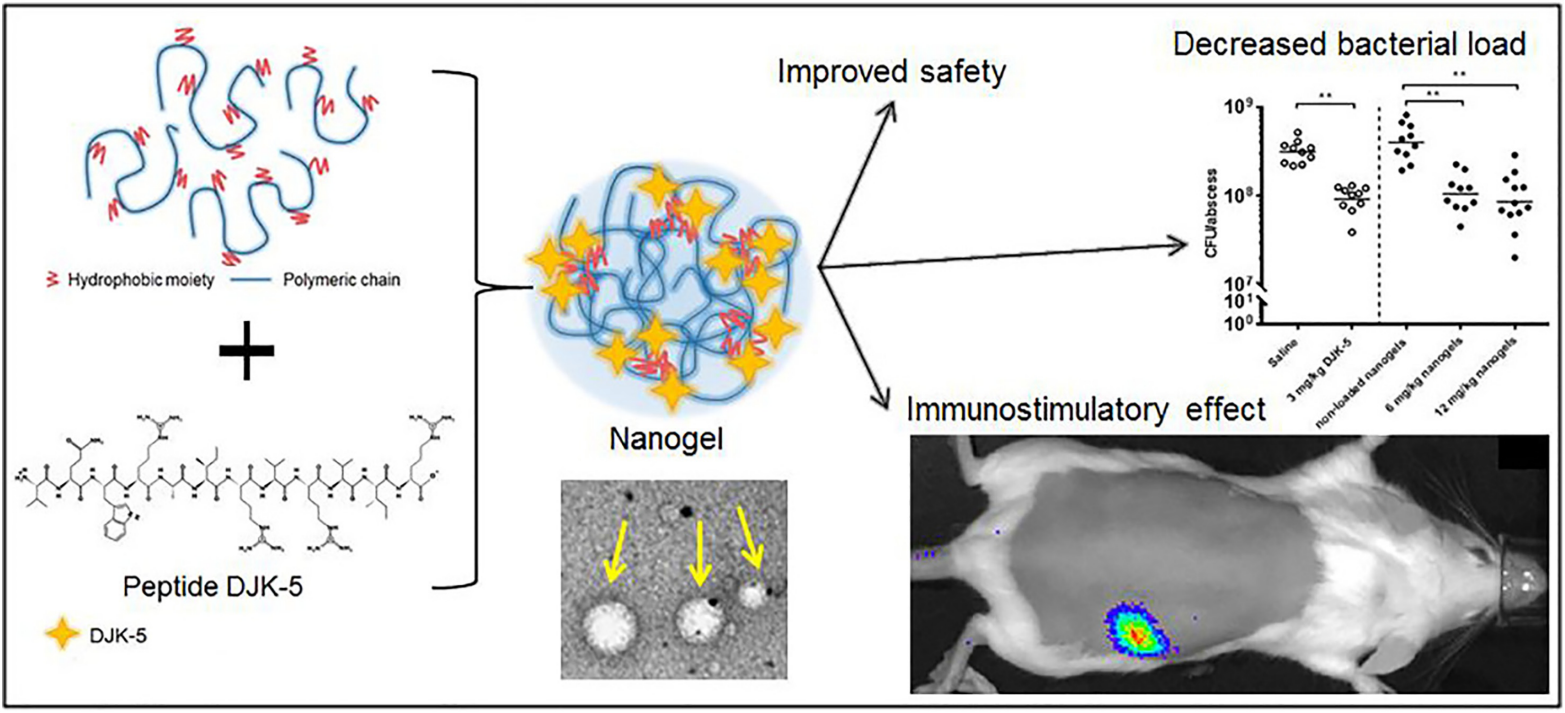
Schematic representation of antibiofilm peptide, DJK‐5, loaded into HA‐NGs (Kłodzińska, Rahanjam, et al., 2019)

Antimicrobial peptides are molecules that have been identified as among the most promising therapeutic candidates for the treatment of bacterial infections. However, these agents showed low stability and bioavailability as well as a high toxicity profile (Teixeira et al., 2020). Hyaluronic acid‐based nanogels have been studied recently as a promising nanoplatform for the delivery of antimicrobial peptides and peptidomimetics (P. D. Kłodzińska, Rahanjam, et al., 2019; S. N. Kłodzińska et al., 2018; Silva et al., 2016). To this end, Silva et al. (2016) reported HA‐NGs loaded with LLKKK18 peptide to cure pulmonary mycobacterial infections (Figure 6). HA‐NGs were prepared via cross‐linking of HA with a thiolated alkyl chain and produced NGs with a large diameter (533 nm), small PDI (0.1), and high EE% (70%). The biocompatibility of the loaded NGs in bone marrow‐derived macrophages (BMMΦ) was evaluated using two distinct assays. Both assays revealed that loaded NGs had no harmful impact on BMMΦ at concentrations up to 100 μM, which is more than 20‐fold the toxic concentration of free peptide. Furthermore, activated macrophages overexpress the CD44 receptor, allowing infected macrophages to successfully uptake loaded HA‐NGs, resulting in selective targeting of LLKKK18 to mycobacteria that reside in intracellular compartments. In vitro incubation of macrophages with LLKKK18‐loaded NGs reduced *Mycobacterium avium* and *Mycobacterium tuberculosis* intracellular levels. Notably, in vivo studies with LLKKK18‐loaded NGs demonstrated a substantial reduction in infection levels in mice infected with *M. avium* or *M. tuberculosis* (Silva et al., 2016). This study established a strong in vitro–in vivo correlation, thereby validating the potential of LLKKK18‐loaded HA‐NGs to eliminate mycobacteria efficiently. Moreover, HA‐NGs enhanced stability and minimized cytotoxicity, while potentiating peptides targeting the main sites of infection. Continuing the research study into the delivery of antimicrobial peptides and peptidomimetics, Kłodzińska and his group developed HA‐NGs by conjugating HA with octenyl succinic anhydride. In their first study, they explored the ability of the HA‐NGs to enhance the antibacterial activity and biosafety of antimicrobial peptidomimetics (lysine‐based α‐peptide/β‐peptoid). This was the first reported biopolymer NGs incorporating antimicrobial peptidomimetics. The NGs were prepared using the microfluidic chip technique. Design of Experiments (DoE) was used to assess the optimal parameters for preparation of the NGs; accordingly, 12 formulations were prepared (F1–F12). F10 was the most encouraging in this study with size, zeta potential (ZP), and EE% of 175 nm, −16 mV, and 88%, respectively. The HA‐NGs encapsulating the peptidomimetic LBP‐3 exhibited no hemolysis and reduced LBP‐3 cytotoxicity in hepatocytes (HepG2 cells) when compared to free peptidomimetic, whereas the MIC and minimum biofilm inhibitory concentration (MBIC) values of LBP‐3 against *P. aeruginosa* remained unchanged. Only F10 exhibited the potential to delay the regrowth of *P. aeruginosa* (Kłodzińska et al., 2018). Despite the significant enhancements, this study lacked investigations into morphology, the peptidomimetic release from the NGs, and in vivo studies, which would have further validated the ability of HA‐NGs to effectively deliver LBL‐3 and other peptidomimetics. Another peptide‐loaded HA‐NG was formulated by the same group, this time focusing mainly on improving peptide biosafety. Kłodzińska et al. (2018) evaluated the ability of HA‐NGs to encapsulate the antibiofilm peptide DJK‐5 with the overall goal of improving the in vivo biosafety of DJK‐5s (Figure 7). For this purpose, NGs with sizes ranging from 174 to 194 nm encapsulating 33%–60% of DJK‐5 were prepared. This EE% was relatively low compared to their prior LBP‐loaded HA‐NGs, where the EE% reached 88%. The in vitro release studies showed that 80% of the loaded peptide was released within 5 h. The in vivo biosafety of DJK‐5 and DJK‐loaded NGs was evaluated using a *P. aeruginosa* abscess model following both IV and subcutaneous administration. Encapsulation of DJK‐5 in HA‐NG reduced its toxicity by 2‐fold following IV administration, and by 4‐fold after subcutaneous injections, compared to the free peptide, without compromising the anti‐abscess efficacy of DJK‐5 (P. D. Kłodzińska, Rahanjam, et al., 2019). In comparison to their previous study, all essential physiochemical and biological characterization stages (in vitro and in vivo) were carried out perfectly in this study, hence supporting the use of HA‐NGs to improve the safety of antibacterial and anti‐biofilm peptides. Taken together, the findings of these studies support the application of HA‐NGs to enhance antimicrobial peptides and peptidomimetics delivery owing to their ability to improve antibiofilm activity, antibiotic localization on‐site, and to eliminate the cytotoxicity of loaded drugs. Finally, various tailored HA‐NGs and formulation conditions can be explored to determine the most appropriate delivery system for peptide and peptidomimetics and allow their efficient use in the clinic.

In addition to the above‐mentioned applications of HA‐NGs, HA‐NGs have been also formulated without drug loading. Since *S. aureus* is able to persist within keratinocytes and phagocytic cells being protected from both the immune system and a variety of antibacterials, to overcome these obstacles, nano‐formulations that enable targeted therapies against intracellular *S. aureus* might be developed. In this regard, Montanari et al. (2020) formulated blank HA‐NGs (with no drug) to investigate the biodistribution and intracellular localization of HA‐NGs (compared to HA) following intravenous (IV) injections (in mice) and topical administrations (in ex vivo human skin). All HA and NGs used in this study were labeled using rhodamine B isothiocyanate without loading of any drug. Following IV injection, HA accumulated considerably in the skin, which corroborates the findings of Pedrosa et al. (2017), who showed that HA‐NGs exhibited high accumulation in the skin but with a blood clearance rate slower than HA (Pedrosa et al., 2017). Furthermore, after topical administration in an ex vivo human skin model, neither HA nor its NGs penetrated the intact skin. In contrast, in both barrier‐disrupted skin and mechanically produced wounds, HA was taken up by dermis cells in a higher percentage than HA‐NGs; however, in both cases, the uptake is mediated by the CD44 receptor. Then, using human dermal fibroblasts and murine macrophages, they investigate the intracellular fate of HA and its NGs in dermal cells. Interestingly, the data obtained suggest the potential of both HA and HA‐NGs to accumulate in dermal cell lysosomes, which is in accordance with their previous work (Montanari et al., 2018, 2020).

From all the papers discussed in this subsection, the loaded molecule, molecular weight of HA, cross‐linked hydrophobic moiety, targeted bacteria, and in vitro/in vivo status information were extracted and then presented in Table 1. So far, only four types of hydrophobic moieties have been used to formulate HA‐NGs. Therefore, in the future, other hydrophobic moieties such as methacrylate molecules, fatty acids, acetic anhydride, as well as active antibacterial compounds, could be investigated. In summary, the findings presented in this subsection confirm the potential of HA‐NGs as a promising nanosystem for antibacterial delivery, with the capability to offer selective targeting while also improving bioavailability, penetration, and antibacterial activity. Moreover, HA‐NGs provide controlled release behavior and reduce the toxicity of loaded compounds.

**TABLE 1:** Summary of HA‐NGs for antibacterial delivery

Antibacterial agents	Molecular weight of HA (KDa)	Cross‐linked hydrophobic moiety	Targeted bacteria	Key characterization methods	Proof of efficacy status	References
Levofloxacin	200	Cholesterol	*S. aureus* and *P. aeruginosa*	Size and PDI EE% Stability studies	In vitro (MIC and intracellular HeLa‐infected cell model)	(Montanari et al., 2014)
Gentamicin or levofloxacin	200	Cholesterol	*S. aureus*	Size, PDI and ZP EE% and DLC% MTT assay	In vitro (MIC, MBC, intracellular HaCaT cell infection model, cellular uptake studies)	(Montanari et al., 2018)
Azithromycin	50	Octenyl succinic anhydride	*P. aeruginosa*	Size, PDI and ZP EE% MTT assay	In vitro (MIC, antibiofilm, motility assay)	(S. N. Kłodzińska, Wan, et al., 2019)
Enrofloxacin	Not mentioned	Chitosan	*S. aureus*	Size, PDI and ZP EE% and DLC% SEM DSC, XDR and FTIR	In vitro (Zone of inhibition, MIC and TKA)	(Yuda Liu et al., 2021)
LLKKK18 peptide	7.46	11‐amino‐1‐undecanethiol	*M. avium* and *M. tuberculosis*	Size, PDI and ZP EE % MTT assay	In vivo (Mice infected with M. avium or M. tuberculosis)	(Silva et al., 2016)
Peptidomimetic (LBL‐3)	50	Octenyl succinic anhydride	*P. aeruginosa*	Size, PDI and ZP EE% and DLC% MTT and hemolysis assays	In vitro (MIC, MBIC and TKA)	(S. N. Kłodzińska et al., 2018)
DJK‐5 peptide	50	Octenyl succinic anhydride	*P. aeruginosa*	Size, PDI and ZP TEM EE % circular dichroism	In vivo (Murine abscess model)	(Kłodzińska, Rahanjam, et al., 2019)
No drug	200	Cholesterol	*S. aureus*	–	In vivo and ex‐vivo wound model	(Montanari et al., 2020)

Abbreviations: DLC, drug loading capacity %; DSC, differential scanning calorimetry; EE%, entrapment efficiency %; FTIR, Fourier transform infrared; PDI, polydispersity index; SEM, scanning electron microscopy; TEM, transmission electron microscopy; XDR, x‐ray diffraction; ZP, zeta potential.

### 3.2 Hyaluronic acid‐ion complex NPs

Owing to its anionic nature, HA tends to form ion complex NPs with cationic small molecules or polymers through polyelectrolyte complexation, which is based on the ability of polyelectrolytes to cross‐link in the presence of counter ions. This is due to the fact that coupling of complementary ionic building blocks could result in molecular configurations capable of self‐assembling into well‐organized nanostructures (S. N. Kłodzińska & Nielsen, 2021).

**FIGURE 8:**
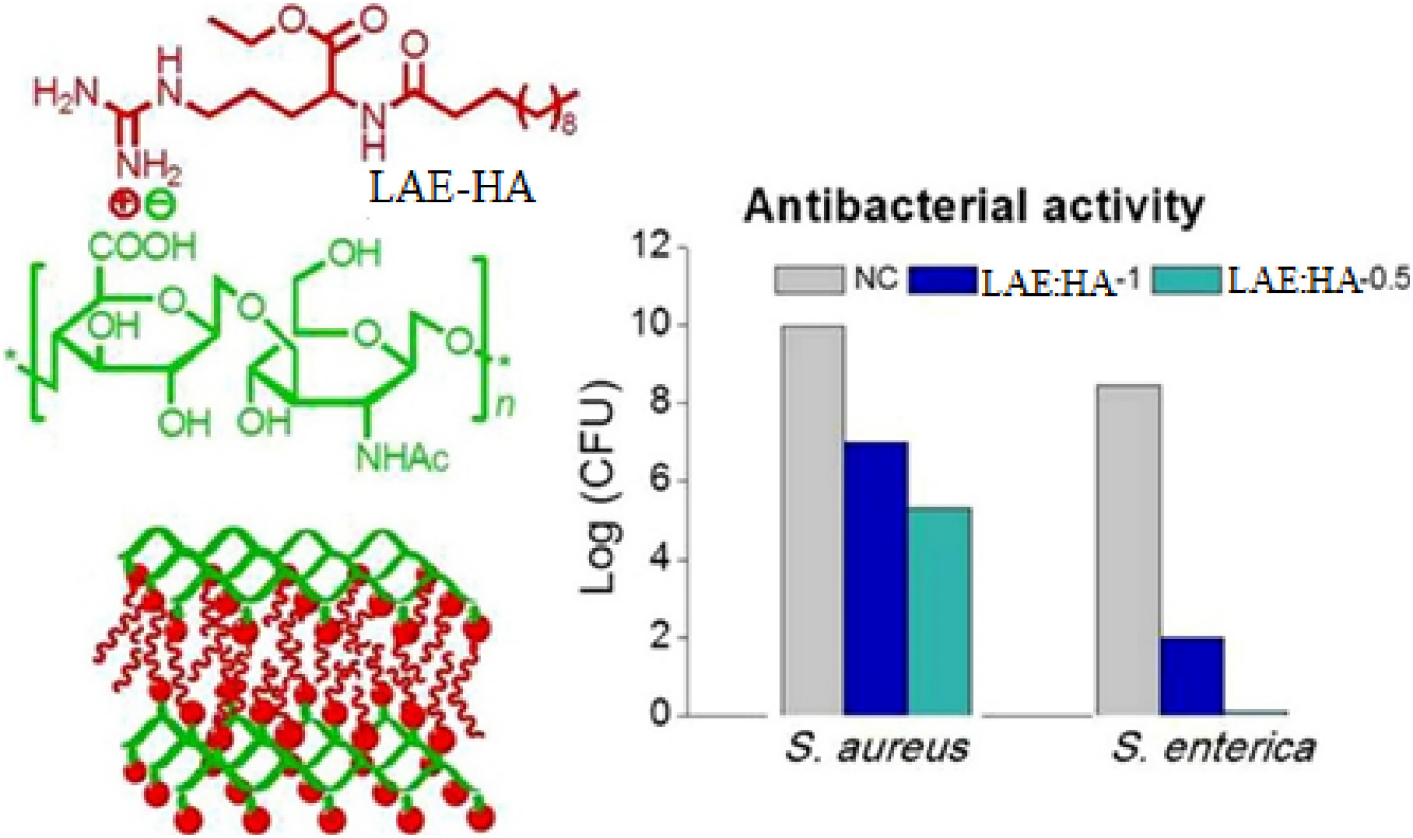
Scheme illustrating the ionic complexation of LAE‐HA and its antibacterial activity (Gamarra et al., 2018)

To date, two such nanosystems have been reported in the literature for potential applications in the prevention as well as therapy of bacterial infections. Electrostatic assembly of HA with an arginine‐based compound, called ethyl‐α‐N‐lauroyl‐l‐arginate hydrochloride (LAE), to build antimicrobial nanofilms with potential application in food preservation and designing of antiseptic medical devices has been reported by Gamarra et al. (2018) (Figure 8). Thin films prepared from HA‐LAE by casting exhibited a smectic‐like structure based on an ordered arrangement of LAE and HA layers with a nanometric periodicity of 3.8–3.9 nm. In such systems, HA acts as a matrix for holding and regulating the antimicrobial release from LAE, therefore reducing its potential toxicity and prolonging its activity time compared to the neat LAE. Thereafter, the rate of LAE release from the nanofilm was studied at two pH levels (5.5 and 7.4). Within 4–8 h of incubation, the dissociation equilibrium was reached. At that time, the concentration of LAE was much greater at pH 7.4 than at pH 5.5, and the quantity of free LAE accumulated significantly increased with the LAE: HA ratio in the nanofilm. The antibacterial activity of LAE‐HA at pH 7.4 was explored using the gram‐positive (*L. monocytogenes* and *S. aureus*) and gram‐negative (*S. enterica* and *E. coli*) bacteria. The results indicated significant bactericide activity against both gram‐positive and gram‐negative bacteria, with the highest activity observed against *S. enterica*. Although further in vitro biocompatibility and in vivo antibacterial studies are required to support their hypothesis, the findings of this research demonstrate that HA has the potential to form arginine‐based nanofilms with inherent antibacterial activity as an alternative antiseptic and preservation agent.

Current TB therapies yield low drug loading and/or accumulation in the lung alveoli. Therefore, Mukhtar et al. (2020) developed dry powder inhalers containing CS/thiolated CS (TC) in conjugation with HA as an innovative drug delivery strategy that will allow the antibacterial agent to penetrate deeper lung tissues. The ionic gelation approach was used to produce CS‐HA and TC‐HA NPs, which were subsequently loaded with isoniazid (INH). Several formulations were developed using DoE, and the average particle size of optimized formulations was 300.2 nm and 342.1 nm with EE% of 90.23% and 92.58% for CS‐HA and TC‐HA NPs, respectively. The NPs carried a cationic ZP, making them favorable to target the negatively charged surface of macrophages. Additionally, incorporating HA into NPs enables targeting of macrophage CD44 surface receptors, therefore activating a synergistic strategy. Later the in vitro drug release of both NPs, a controlled release pattern was revealed over time, with approximately more than 50% of INH released from both. The MTT cell viability assay was carried out on adenocarcinoma human alveolar basal epithelial (A549) cells. Isoniazid‐loaded NPs were found to be biocompatible with the A549 cell line and had no cytotoxicity at concentrations ranging from 0.1 to 0.5 mg/ml. Moreover, the in vitro drug accumulation followed by Andersen Cascade measurement was promising and aligned with the in silico particle deposition model for TB. Both formulations exhibited a greater amount of particle accumulation in the alveolar area, which was considered the site of action. Collectively, the results of this study proposed a novel HA‐NC that actively delivers anti‐TB medication to the site of action while also improving the pulmonary drug deposition; however, antibacterial studies are still required to confirm the antibacterial effects of this nanosystem (Mukhtar et al., 2020).

The above‐mentioned data demonstrates that the complexation of cationic molecules with HA for targeting bacteria can lead to controlled drug release and consequent inhibition of bacterial growth in infected areas (Gamarra et al., 2018; Mukhtar et al., 2020).

### 3.3 Hyaluronic acid‐polymersomes

In recent decades, polymersomes have garnered remarkable attention for antibacterial delivery owing to their colloidal stability, flexible membrane characteristics, and capacity to encapsulate or integrate a wide variety of drugs and molecules. Polymersomes are defined as self‐assembling nano‐vesicles composed of amphiphilic block copolymers. Along with its application in antibacterial delivery, polymersomes are also efficiently utilized to encapsulate therapeutic molecules such as anti‐inflammatory, anticancer, antifungal, as well as DNA and RNA fragments either in the aqueous core or integrated into their membrane (J. S. Lee & Feijen, 2012; A. K. Sharma et al., 2020).

The application of HA‐based polymersomes as diagnostic tools is recognized as a popular area of research (Matoori & Leroux, 2020). In this regard, Haas et al. (2015) developed smart enzyme‐sensitive polymersomes to detect pathogenic bacteria such as *S. aureus* in wounds and burn environments. They describe a novel HAase‐responsive amphiphilic block copolymer system based on HA and polycaprolactone (PCL). These polymersomes were constructed using an inversed solvent‐shift technique using chloroform and water followed by loading with various fluorescent dyes and antibacterial agents, resulting in HA‐PCL assemblies varying in size from 50 to 400 nm, depending on the loaded molecules. The polymersomes, as demonstrated by two different imaging microscopes, are vesicular in nature. In the presence of HAase, the in vitro release of the loaded molecule was studied using the dynamic light scattering technique; a reduction in diameter was observed as the reaction time increased, indicating that the polymersomes were successfully degraded by HAase. Additionally, when loaded with a fluorogenic dye, fluorescence spectroscopy revealed a spontaneous light‐up in the presence of HAase and chymotrypsin. However, the degradation of the HA‐PCL vesicles was studied using bovine HAase instead of bacterial HAase, which are different. This work supports the potential of HA‐PCL polymersomes as a smart indicator system for identifying harmful bacteria in wounds. This approach may be exploited to produce innovative detective and therapeutic HA‐polymersomes targeting various HAase‐producing bacteria (Haas et al., 2015).

**FIGURE 9:**
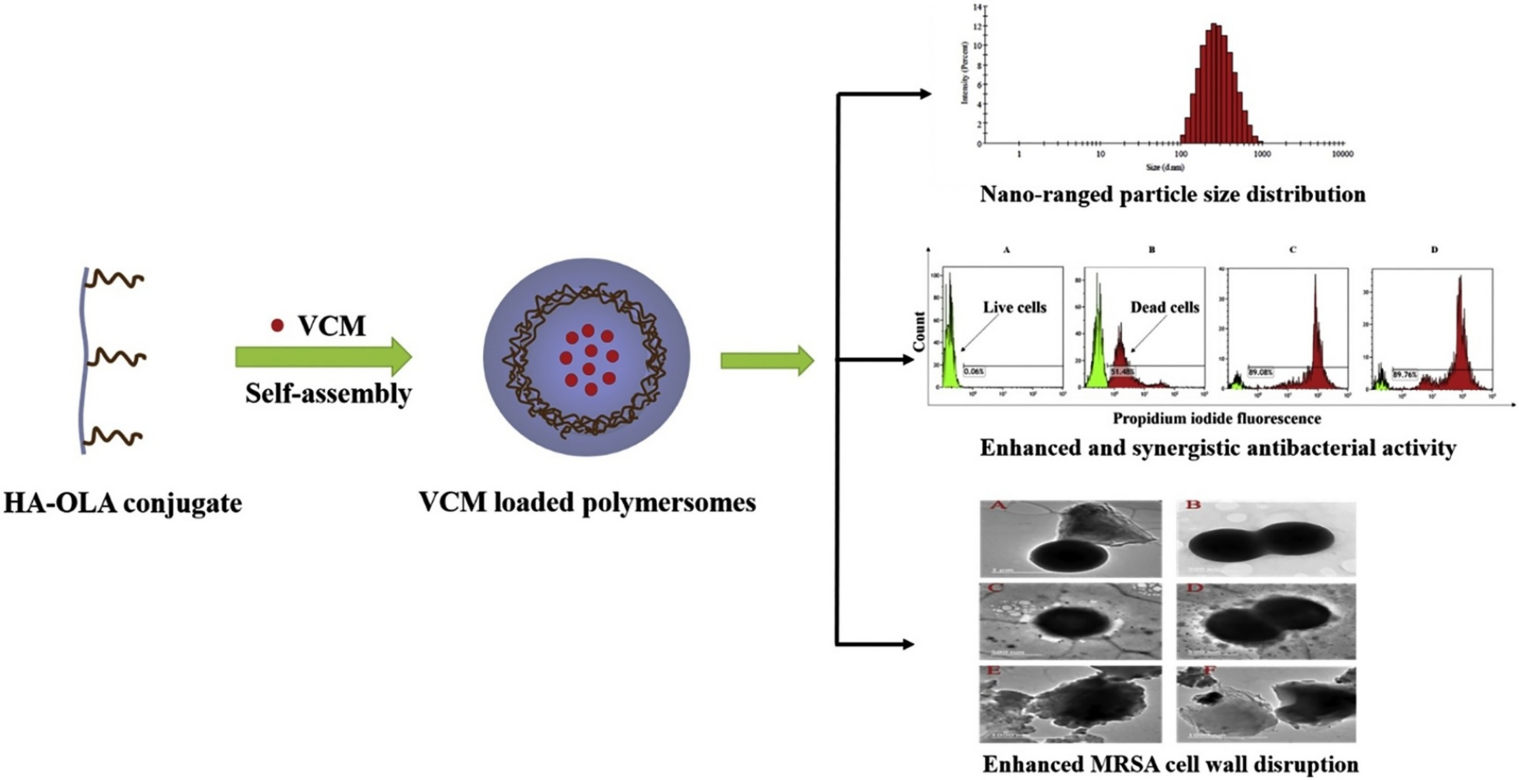
Scheme illustrating formulation of HA‐OLA polymersomes to eradicate MRSA infection (Walvekar et al., 2019)

In addition to the above‐mentioned application in infection diagnosis, HA‐polymersomes have been recently explored for antibacterial delivery. For this purpose, our research group developed novel HA‐oleylamine (HA‐OLA) polymersomes to enhance the antibacterial activity of vancomycin (VCM) against MRSA (Figure 9). Following the synthesis of novel HA‐OLA, the biosafety of the conjugates was confirmed using the MTT assay on three different human cell lines: embryonic kidney cells, cervix adenocarcinoma cells, and human breast adenocarcinoma cells. A probe ultrasonication technique was used to prepare the polymersomes, which were subsequently loaded with VCM. The optimized VCM‐loaded HA‐OLA polymersomes (VCM‐PS6) had an average diameter of less than 250 nm and an EE% of 43.12%. These polymersomes showed slow and sustained release of VCM over 3 days. The in vitro MIC studies against MRSA showed that VCM‐PS6 had fourfold the activity of bare VCM. Furthermore, the synergistic action of VCM and HA‐OLA against MRSA was reported. Additionally, when samples were treated at an MIC of 1.95 g/ml, flow cytometry demonstrated that VCM‐PS6 resulted in 1.8‐fold more dead MRSA cells than free VCM. A bacterial membrane rupture test revealed that VCM‐loaded polymersomes were more effective in disrupting MRSA membranes than free VCM. These findings suggest that VCM‐PS6 was more effective against MRSA than free VCM in all antibacterial tests. In summary, these findings confirm that HA‐OLA polymersomes have the potential to be promising antibacterial nanosystems to tackle resistant bacterial strains (Walvekar et al., 2019).

Of the HA‐NPs studied, and to the best of our knowledge, only the abovementioned polymersomes have been designed, constructed, and studied for their potential in antibacterial therapy. These polymersomes have considerable therapeutic and diagnostic potential for bacterial infections, which can be significantly increased by mimicking the natural substrate of B‐HAase, thus enabling the self‐regulated release of antimicrobials. Accordingly, there is a gap in research for designing innovative multifunctional HA‐based polymersomes for dual detection and treatment of HAase‐producing microorganisms.

### 3.4 Other hyaluronic acid‐based nanocarriers

In addition to all the HA‐NPs mentioned above, others have been reported to either eradicate or prevent bacterial infections. These include HA‐based micelles, nanocapsules, nanoplexes, nanofibers (NFs), and nanofilms (Baier et al., 2013; Gao et al., 2014; Hussein et al., 2020; Montanari et al., 2016).

Nanometer‐sized HA systems can selectively deliver antibacterial drugs into infection sites via interaction with overexpressed CD44 receptors in certain cells, such as macrophages, which allow effective and enhanced localization of loaded antibacterial drugs in the site of infection. For this purpose, two studies have reported HA‐NPs for targeted delivery of antibacterial agents, namely rifampicin (RIF) and tannic acid (TA), to eradicate intracellular bacterial infections (Gao et al., 2014; Montanari et al., 2016). Gao et al. (2014) presented a TB‐targeted drug delivery system based on HA‐functionalized micelles loaded with RIF; a drug widely used for the treatment of TB. Tocopherol succinate was grafted onto HA to generate a hydrophobic moiety enabling self‐assembly. They synthesized an HA‐tocopherol succinate conjugate (HA‐TS), which self‐assembled into HA‐micelles with average particle diameter ranging from 212 to 294.6 nm. The RIF was incorporated with high drug loading capacity exceeding 70%. The in vitro release studies conducted in two different environments (PBS at pH 7.4 and acetate buffer at pH 5.2) revealed the sustained release of RIF at both pH levels; however, a slower release rate of RIF was observed in acidic media compared to the release rate at physiologic pH. The in vitro biocompatibility on murine alveolar macrophage (MH‐S cells) was examined using the MTT assay. The results showed that the loaded micelles exhibited slightly higher cytotoxicity to MH‐S cells compared to free RIF within the first 2 days, while no apparent cytotoxicity was observed for blank HA‐TS micelles with concentrations between 25 and 250 μg/ml. As expected, the loaded micelles were more efficiently taken up by MH‐S cells than the free drug. This uptake occurred via phagocytosis and CD44‐mediated endocytosis, and it was time‐ and dose‐dependent. Additionally, the HA‐TS micelles may promote the release of pro‐inflammatory cytokines, which could improve the antibacterial action of RIF. In another study, Montanari et al. (2016) designed and synthesized HA‐aminophenyl (HA‐APBA) conjugated NPs with a pH‐cleavable linkage for smart delivery of TA to intracellular infection sites. A reaction between HA‐APBA and TA yielded NPs with a size smaller than 400 nm, which was dependent on the molecular weight of HA. The boronate ester bond made the NPs stable at physiological pH, while hydrolyzing in an acidic environment allowed TA release within endosomal compartments. The presence of HA enhanced the selectivity of these NPs toward bacteria that colonize within macrophages. Therefore, RAW 246.7 macrophages were used as the model to assess the cytotoxicity of the NPs. The results indicated that HA‐APBA alone exhibited slight cytotoxicity, TA significantly reduced cell viability at 56 μg/ml only when combined with AA, but neither AA nor TA appeared to have any impact when used alone. The loaded NPs exhibited similar toxicity to the TA‐AA combination. Finally, three bacterial models were employed in the antibacterial study: *S. aureus*, MRSA, and *E. coli*. Both the loaded NPs and TA had no significant action against *E. coli*, while activity against sensitive and resistant *S. aureus* were significantly greater. Interestingly, the NPs demonstrated nearly comparable antibacterial activity to the TA/ascorbic acid combination, confirming that the boronate complexation retains the reduced (catechol) form of TA and that the NPs indeed release this active form of TA in an acidic medium (Montanari et al., 2016). The outcomes of these two studies confirm the potential of HA‐NPs to eradicate intracellular bacteria; however, in vivo antibacterial studies of both nanocarriers are required to validate their theory.

Hyaluronic acid‐NPs can also act as carriers for the purpose of wound dressing and tissues regeneration. Wound dressing has been widely studied in order to develop an “optimal” technique that promotes rapid recovery while minimizing scarring. For this reason, HA‐NPs, with their diverse physicochemical properties, are a promising research domain for wound dressing and healing (Prajapati & Maheriya, 2019). In this regard, Baier et al. (2013) developed novel HA‐based nanocapsules encapsulating an antibacterial agent that cleaves specifically in the presence of HAase. The inverse mini‐emulsion approach was used to prepare stable cross‐linked HA‐based nanocapsules loaded with polyhexanide (PH). The obtained nanocapsules had diameters of 360–380 nm and a negative surface. Furthermore, the release of the entrapped PH was quantified by measuring the absorbance of the model dye that was released in response to HAase. As expected, control nanocapsules composed of starch derivative or only PH as a shell material did not exhibit any enzyme‐responsive release. The in vitro antibacterial studies in the presence of *S. aureus* and *E. coli* revealed that both the PH containing HA‐based (HA‐PH‐NCs) and the PH‐based nanocapsules (PH‐NCs) had antibacterial activity. Additionally, these nanocapsules were found to be bactericidal against both *S. aureus* and *E. coli*. However, the MIC for *S. aureus* was lower, reaching 62.5 μg/ml for both nanocapsules, while their activity against *E. coli* was significantly reduced with MIC values of 250 and 125 μg/ml for HA‐PH‐NCs and PH‐NCs, respectively. This is explained by the capacity of *S. aureus* to generate and release HAase, which cleaves HA and promotes bacterial dissemination (Baier et al., 2013). Using another strategy for wound healing, Hussein et al. (2020) reported novel l‐arginine‐loaded HA‐based NFs as a bioactive wound dressing with inherent wound healing properties. To address the poor mechanical properties of HA‐based NFs, they strengthened them by including cellulose nanocrystals (CNC) as a bio‐composite, which were then loaded with l‐arginine. An electrospinning technique was used to fabricate the HA‐based NFs. Thereafter, HA‐based NFs showed round‐shaped nanoparticles with an average size of 120 nm. It was observed that this size fell to 60 nm after incorporation of the CNC, then increased again to 100 nm after incorporation of l‐arginine. This result was in accordance with Reesi et al., who observed that the size of l‐arginine–loaded lignin NFs increased from ~20–50 nm to ~100–250 nm (Reesi et al., 2018). The loaded HA‐CNC NFs (PVA/HA/CNC/L‐arginine NFs) showed slow‐release behavior, which led to a sustained elevation in the level of arginine in plasma, thus reducing its adverse effects. Interestingly, the slow release of arginine was found to be a trigger for nitric oxide (NO) signaling, thus accelerating wound closure. Using normal human skin melanocyte (HFB‐4) and lung fibroblast (WI38) cell lines, in vitro bio‐evaluation of NFs revealed that PVA/HA/CNC/l‐arginine NFs displayed favorable hemocompatibility, elevated protein adsorption, and excellent proliferation and adhesive capability on HFB‐4 cells. Regarding the antibacterial studies, PVA/HA/CNCs/l‐arginine demonstrated acceptable antibacterial action, particularly against *Klebsiella pneumonia*, a common acute pathogen that causes skin infection (Hussein et al., 2020). Overall, the data presented in this paragraph confirms that such functionalization (using HA) can represent a robust technique for developing multifunctional HA‐nanosystems for wound dressing and infection control.

Infections associated with biomedical devices contribute significantly to the growing problem of nosocomial infections, imposing a considerable societal and economic impact (Khan et al., 2017). Therefore, a HA‐nanofilm was developed to reduce this bioburden. Hernandez‐Montelongo et al. (2016) described the optimal fabrication of layer‐by‐layer HA‐CS nanofilms (HA‐CS) as an antibacterial coating technique for preventing nosocomial infections from biomedical devices. Hyaluronic acid created a soft, well‐hydrated, nontoxic coating for this nanosystem, whereas CS demonstrated antibacterial properties. The optimization of HA‐CS nanofilm synthesis was based on altering the pH values of the biopolymer stem solutions and, as a result, the degree of ionization. Moreover, by increasing the number of HA‐CS bilayers, the surface density of amino groups, which is associated with antibacterial activity, was enhanced. The antibacterial activity of HA‐CS nanofilms was then investigated against *S. aureus* and *P. aeruginosa* using the spread plate counting method. The HA‐nanofilms demonstrated promising results against *S. aureus*, which was highly sensitive to the surface of nanofilms fabricated at various pH values compared to the control sample (silicon substrate). This sensitivity was attributed to the free ammonium group of CS on the top HA‐CS layer of the nanofilms. However, when *P. aeruginosa* cultures were cultured for 4 h at pH 4.5 and pH 3, the HA‐CS nanofilms had no significant antibacterial action, even though the bacteria were grown at slightly lower rates when compared to the control. Thus, HA‐CS nanofilms in the optimized conditions of synthesis shown here are an excellent alternative as antibacterial surfaces against *S. aureus*. Nevertheless, their use against *P. aeruginosa* would require the addition of antimicrobial agent, such as bioactive peptides, silver nanoparticles, and other suitable biocides (Hernandez‐Montelongo et al., 2016).

The outcomes of all the studies mentioned in this section validate the potential application of HA as a promising polymer to formulate HA‐NPs for preventing and eradicating bacterial infections. These NPs can enhance the localization of antibacterial agents by providing smart targeted antibacterial therapy, lowering adverse effects of loaded drugs, and regulating the release of the loaded antibacterial agents.

## 4 HYALURONIC ACID‐COATED NANOCARRIERS

The surface of nanocarriers can be functionalized with a variety of polymers depending on the surface characteristics of nanocarriers (Almalik et al., 2017; Frank et al., 2020; Muddineti et al., 2015). Coating nanocarriers with HA may improve their biological characteristics and biocompatibility, boost their antibacterial activity, and enable controlled site‐specific drug release. Additionally, coating with HA can facilitate the ability of nanocarriers to evade immune cell detection and uptake by the reticuloendothelial system, while also significantly improving target recognition and localization. Therefore, HA‐coated nanocarriers have been widely applied for targeted drug delivery in the treatment and diagnosis of cancer. However, the use of HA to coat antibacterial‐loaded NPs is still in its infancy (Arora et al., 2016; K. Kim et al., 2019; Osman et al., 2021; Sakurai & Harashima, 2019). In this section, we discuss and critically elaborate on the antibacterial nanocarriers that have been surface‐modified using HA, which are summarized in Table 2. We have systematically categorized these nanocarriers according to the function of the HA layer into (i) CD44 targeting nanocarriers; (ii) HAase‐responsive nanocarriers; and (iii) others.

**TABLE 2:** Hyaluronic acid‐coated nanocarriers tested for their antibacterial activity

Nanocarrier	Cargo	Size (nm)	Targeted bacteria	Responsive stimuli	Therapy strategy	Proof of efficacy statues	References
TZH	Tetracycline	500	*S. aureus* *Salmonella typhi*	Acidic environment	Chemotherapy	In vivo	(X. Zhang et al., 2019)
ZVH	Vancomycin	389	MRSA	Acidic environment	Chemotherapy	In vivo	(Yinyin Liu et al., 2020)
GOD/Ag@ZIF‐HA	AgNPs	200	*S. aureus* *E. coli*	H_2_O_2_ from glycolysis	Chemotherapy	In vivo	(Yaojia Li et al., 2021)
HA‐P(Au/Ag)	Ag^+^	138	MDR‐AB	Nonresponsive	Chemotherapy, Photothermal	In vivo	(Yang et al., 2020)
MSN‐Lys‐HA‐PGMA	Amoxicillin Lysozyme	175	*S. aureus*	B‐HAase	Chemotherapy	In vivo	(Wu et al., 2015)
Ab@S‐HA@MMSNs	Vancomycin	240	*S. aureus*	B‐HAase	Diagnosis, Chemotherapy	In vitro	(Xu et al., 2019)
GO‐HA‐AgNPs	Ag^+^	–	*S. aureus*	B‐HAase	Chemotherapy Photothermal	In vivo	(Ran et al., 2017)
AA@Ru@HA‐MoS2	Ascorbic acid, Ciprofloxacin	206	*S. aureus* *P. aeruginosa*	B‐HAase	Chemotherapy Photothermal	In vivo	(Yanan Liu et al., 2019)
HA‐AgNPs	Ag^+^	–	*S. aureus* *E. coli*	Nonresponsive	Chemotherapy	In vitro	(Abdel‐Mohsen et al., 2013)
HA‐AC NPs	Aminocellulose	50	*E. coli* *S. aureus*	Nonresponsive	Chemotherapy	In vitro	(Ivanova et al., 2018)

### 4.1 Hyaluronic acid‐coated nanocarriers targeting CD44 receptors

The primary receptor for HA, which is CD44, is overexpressed in a subset of cells, such as the majority of cancer cells and activated inflammatory cells (including macrophages). This can boost the selectivity of HA‐NPs through CD44‐mediated uptake, allowing HA to serve as a homing device (C. Chen et al., 2018; J. H. Kim et al., 2018; Pandey et al., 2017). Among the several HA‐coated nanocarriers, inorganic NPs are gaining considerable interest for antibacterial delivery due to their high stability, simplicity of functionalization, and inertness. Apart from improving the targeting of these nanocarriers, HA also regulates and controls drug release and increases the colloidal stability (Y. Chen, Chen, & Shi, 2014; Ivanova et al., 2018; Ran et al., 2017; Sekhon & Kamboj, 2010).

**FIGURE 10:**
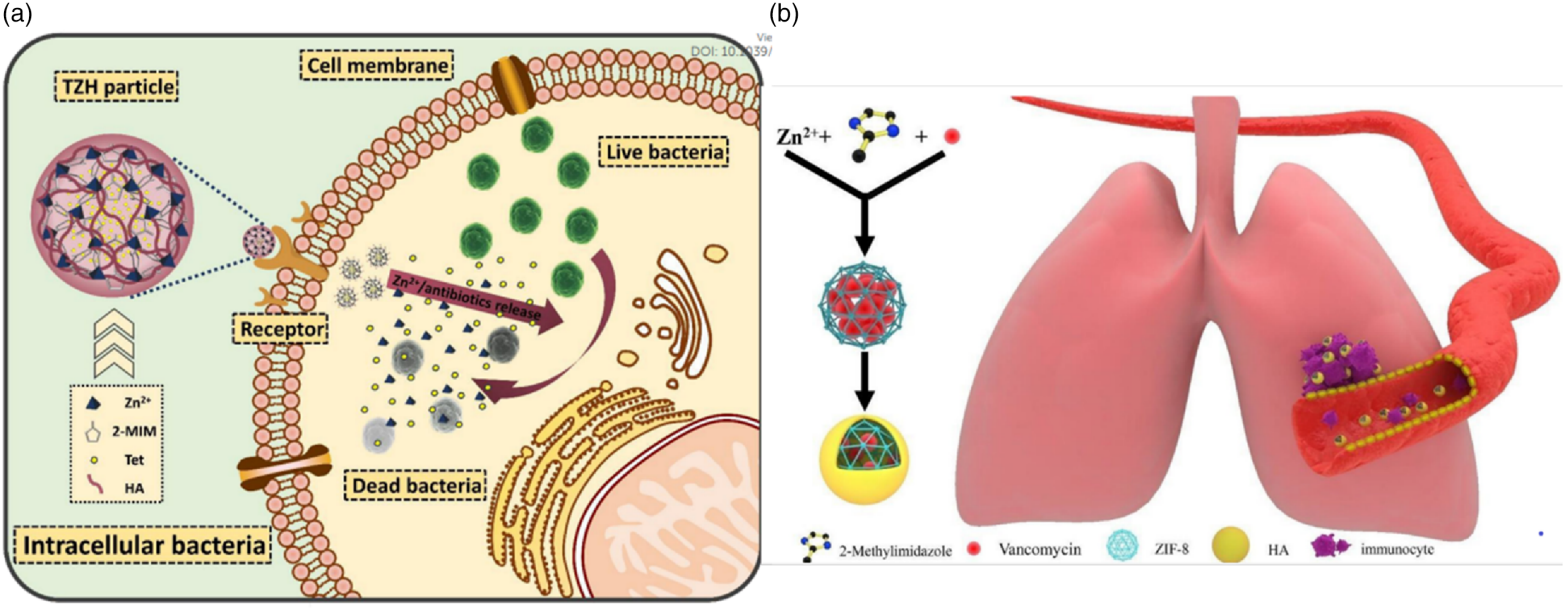
Schematic illustration of (a) tetracycline‐loaded HA‐ZIF8 and (b) VCM‐loaded HA‐ZIF8 targeting intracellular bacteria (Liu et al., 2020; X. Zhang et al., 2019)

Macrophages, the predominant site of intracellular infection, shield intracellular bacteria from the immune cells, therefore limiting the action of antibiotics. These macrophages express CD44 receptors. Thus, modifying nanocarriers with HA enables the development of therapeutic NPs that may actively target these macrophages, enhancing the release and localization of the loaded antibiotics (Jordan et al., 2015; Mattheolabakis et al., 2015; Sánchez et al., 2020). To this end, X. Zhang et al. (2019) and Liu et al. (2020) designed pH‐responsive HA‐coated metal‐organic frameworks (MOFs) loaded with antibiotics for targeted elimination of intracellular bacteria. Zeolitic imidazolate framework‐8 (ZIF‐8) was chosen as an appropriate MOF owing to its acceptable stability, minimal cytotoxicity, and acid sensitivity, which facilitates drug release at the site of infection. The antibiotics were loaded into the ZIF‐8 formed by the self‐assembly of the zinc ion and organic bond via an aqueous phase self‐assembly approach. Then, HA was added to decorate the surface of the nanocarrier via the coordination effect between its carboxylic acid groups and Zn^2+^ in ZIF‐8. In both studies, the nanocarriers can be taken up by cells via an HA‐mediated pathway owing to CD44 receptors on the macrophage surface (Liu et al., 2020; X. Zhang et al., 2019). X. Zhang et al. encapsulated tetracycline in HA‐coated ZIF‐8 to create tetracycline‐loaded HA‐ZIF‐8 (TZH) as shown in (Figure 10a). The in vitro drug release study was carried out at pH 5.5 and 7.4. As expected, both tetracycline and Zn^2+^ were released rapidly from TZH at pH 5.5 compared to the physiological pH. Both in vitro and in vivo antibacterial results demonstrated that ZIF‐8 and tetracycline encapsulated in TZH had a synergistic effect, with an intracellular bacterial eradication rate exceeding 98% at a safe low dose, which was significantly greater than the effect of equal amounts of antibacterial agents alone. Similarly, Liu et al. loaded their HA‐coated ZIF‐8 (HA‐ZIF8) with VCM, and the formed nanocarrier was abbreviated as ZVH (Figure 10b). The release studies were carried out in PBS solutions at pH values of 5.5, 6.5, and 7.4. The results indicated that a larger amount of VCM was released at pH 5.5 than at the other two pH levels. The MTT assay on RAW 264.7 cells displayed slight dose‐dependent cytotoxicity; nevertheless, the formulation was biocompatible at concentrations below 60 μg/ml. Both in vitro and in vivo antibacterial studies confirmed the superiority of VCM loaded in the HA‐ZIF8 compared to the same dose of bare VCM. These results confirmed that TZH and ZVH are unique biosafe nanoplatforms that are able to selectively deliver antibiotics to pathogenic sites and achieve responsive and controllable release, thus improving the therapeutic effect against resistant infections and providing a new platform for further overcoming antibiotic resistance (Liu et al., 2020; X. Zhang et al., 2019). Li et al. (2021) reported another HA‐ZIF8, wherein they elaborated the use of HA‐coated MOFs to deliver silver NPs (AgNPs) rather than conventional antibacterial agents. In this nanosystem, ZIF‐8 accompanied by glucose oxidase was loaded with sliver nanocubes and subsequently coated with HA to enhance their biocompatibility and capability to target CD44. Interestingly, this nanosystem can function as a pool for releasing silver ions (Ag^+^) and tiny Ag NPs (5 nm) from large silver nanocubes (30 nm) in response to bacterial microenvironments. The nanocomposite formed, termed GOD/Ag@ZIF‐HA, had significant antibacterial activity against the studied bacteria with an MIC value of less than 10 μg/ml; this is concomitant with the controlled release profile of Ag^+^ and the small size of AgNPs in the presence of bacteria. Additionally, at the MIC concentration, GOD/Ag@ZIF‐HA inhibited the growth of tested bacteria completely (Li et al., 2021). Even though biocompatibility studies are still outstanding for this research, this study revealed a promising Ag nanoplatform with both size‐switchable and selective targeting ability.

While all the above‐mentioned studies investigated MOFs, a recent study expanded the pool and explored HA‐coated nanocages. Yang et al. (2020) presented gold‐silver hybrid nanocages coated with antimicrobial peptide and HA (HA‐P(Au/Ag)). This study aimed to treat pneumonia by eradicating multidrug‐resistant bacteria‐Acinetobacter baumannii (MDR‐AB). These HA‐P(Au/Ag)s were biosafe with negligible cytotoxicity and hemolytic effects. The functionalization of the surface of the nanocages with HA facilitates the adhesion of HA‐P(Au/Ag) to the bacteria, enabling the loaded antibacterial agents to penetrate and kill the bacteria. Thus, in vitro antibacterial tests revealed that the MIC and MBC values of uncoated nanocages were reduced to 3 and 12 μg/ml, respectively, after coating with HA and the peptide. Furthermore, combining photothermal therapy with the coated nanocages further potentiates their antibacterial activity and entirely eradicates MDR‐AB. Similarly, the in vivo antibacterial studies using a pneumonia mice model revealed that treatment with HA‐P(Au/Ag) dramatically improved the survival rate of tested mice while restoring the normal structure of the alveoli. This study proved that HA‐P(Au/Ag) in combination with photothermal treatment is a viable approach for potentially overcoming MDR‐AB multiple mechanisms (Yang et al., 2020).

Collectively, these findings elucidate that surface coating of nanocarriers with HA for targeting CD44 receptors on the surface of phagocytic cells can improve their uptake and internalization, resulting in targeted drug release and subsequent eradication of bacteria within infected macrophages.

### 4.2 Hyaluronic acid‐coated nanocarriers for hyaluronidase responsiveness

Bacterial infection sites are characterized by acidic pH as well as the presence of numerous virulent enzymes, including B‐HAase. Therefore, exploiting these enzymes for diagnostics, drug targeting, and drug release is remarkably promising (Devnarain et al., 2021). Since HA can be easily degraded by B‐HAase, HA has been widely employed as a targeting and capping agent. As previously reported, many species of bacteria, including *S. aureus*, *Clostridium difficile*, and others, were capable of generating HAase (Baier et al., 2013; Hynes & Walton, 2000). Hence, by taking advantage of this phenomenon, HA (as a coating agent) can be used to detect and/or eradicate these bacteria (Wu et al., 2015; Xu et al., 2019).

**FIGURE 11:**
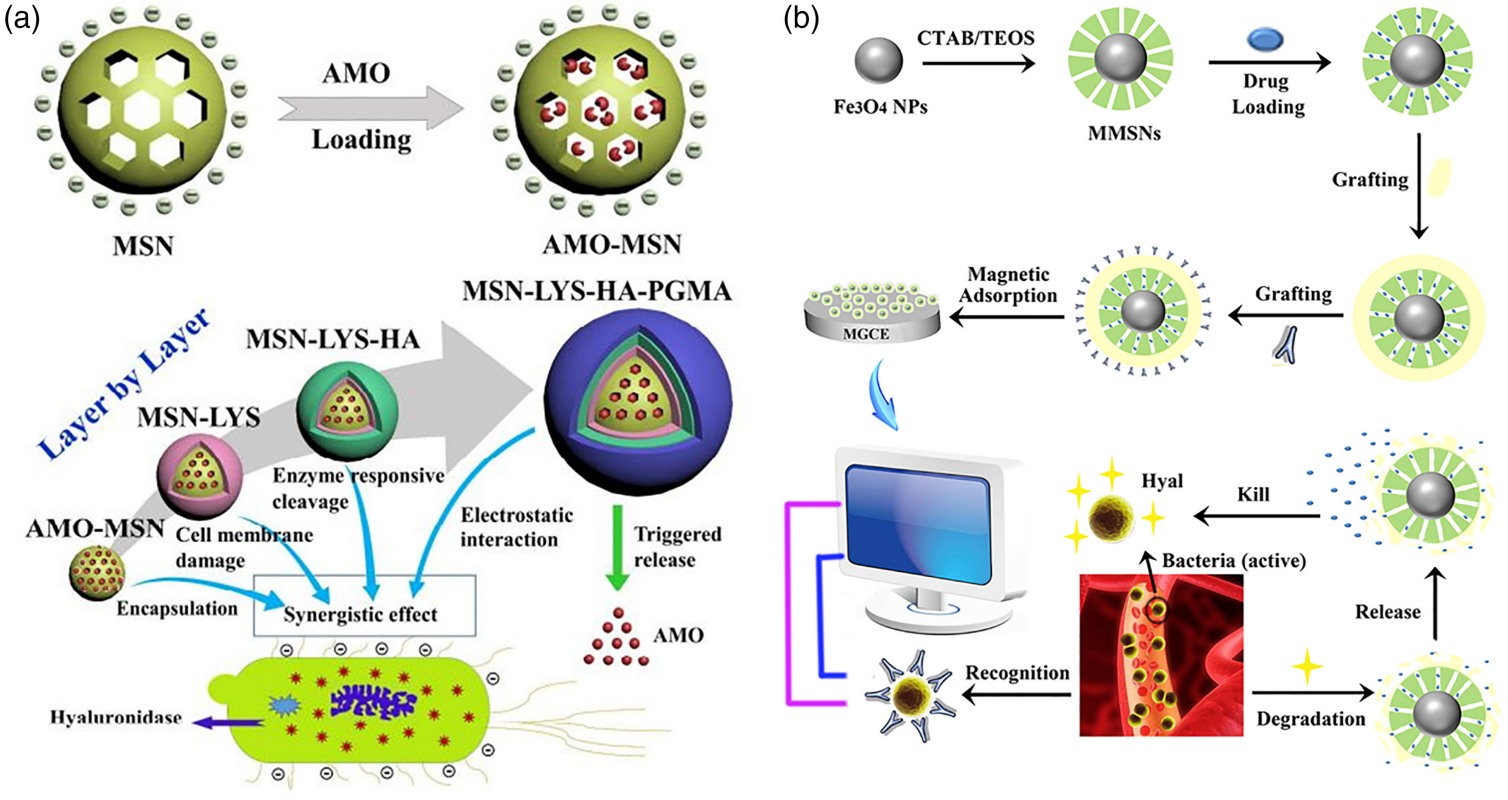
Schematic representation of (a) layer‐by‐layer coating of MSN‐Lys‐HA‐PGMA and the possible antibacterial mechanism for each layer. (b) Synthesis of an “on‐demand” integrated Ab@S‐HA@MMSNs for diagnosis and treatment toward *S. aureus* (Wu et al., 2015; Xu et al., 2019)

In recent years, mesoporous silica nanoparticles (MSNs) have been introduced as a promising drug delivery system. Mesoporous silica nanoparticles have essential parts of nanomedicines owing to their chemical stability, large encapsulation capacity, favorable biosafety, ease of preparation and modification, and tunable pore size (Castillo & Vallet‐Regí, 2021). Thus, coating MSNs with HA could efficiently provide anti‐adhesion properties in physiological fluids, as well as on‐demand drug release in response to B‐HAase (Wu et al., 2015; Xu et al., 2019). In this regard, Wu et al. (2015) explored the potential of layer‐by‐layer coated MSNs to release encapsulated amoxicillin in response to the HAase of *S. aureus*. In this study, amoxicillin, an example of a broad‐spectrum antibiotic, was encapsulated into MSNs and subsequently coated with antimicrobial lysozyme, followed by a second layer of HA, and then a third layer of modified cationic polyglycerol methacrylate (Figure 11). The prepared NPs, abbreviated as MSN‐Lys‐HA‐PGMA, had a diameter of 174 nm and a drug encapsulation capacity of 8.5%. The presence of the HA layer enables enzyme‐responsive release behavior in the presence of B‐HAase, which leads to the enzymolysis of MSN‐Lys‐HA‐PGMA, followed by the release of lysozyme and the loaded amoxicillin at the site of action. The biosafety evaluation of MSN‐Lys‐HA‐PGMA was carried out using the CCK‐8 assay and hemolysis studies. Both attested to the biocompatibility of the coated nanosystem. In vitro antibacterial action was assessed by determining MIC, zone of inhibition, and bacterial cell viability. These studies showed that MSN‐Lys‐HA‐PGMA had higher antibacterial activity than the equal dose of the loaded antibacterial agents alone. However, their efficacy against gram‐negative bacteria was lower due to the presence of the lipopolysaccharide layers, which may inhibit the synergistic action of the lysozyme. Furthermore, the in vivo antibacterial test confirmed the superior activity of the MSN‐Lys‐HA‐PGMA against with a 99.9% inhibition rate, which was consistent with the in vitro findings (Wu et al., 2015).

Magnetic MSN, in contrast to the nonmagnetic MSN discussed above, enables integrated diagnosis and treatment of infection owing to its excellent magnetic property. As a step toward this direction, Xu et al. (2019) reported on‐demand magnetic MSNs for the detection and treatment of *S. aureus*. In this study, magnetic MSNs were loaded with VCM, and then coated with sulfonated HA, followed by functionalization of the surface of magnetic MSNs (Ab@S‐HA@MMSNs) with *S. aureus* antibodies by chemical reaction. Here, sulfonated‐HA, instead of bare HA, was chosen owing to superior anticoagulant and release behavior. The prepared Ab@S‐HA@MMSNs were spherical with a size of 240 nm and a maximum VCM loading capacity of 10.7%; thereafter these NPs were functionalized on the surface of magnetic glassy carbon electrode (MGCE). Therefore, since these NPs have both HA (which exerts anticoagulant action) and *S. aureus* antibodies, they can be directly applied to detect the amount of pathogenic *S. aureus* in whole blood. In addition, with the increased amount of *S. aureus* arriving at the MGCE, the coat of Ab@S‐HA@MMSNs was degraded by B‐HAase leading to the release of the encapsulated VCM and ultimately, the effective eradication of *S. aureus*. At a concentration below 500 μg/ml, Ab@S‐HA@MMSNs were hemocompatible with a hemolysis rate of less than 5%. Owing to the on‐demand controlled release of VCM, the antibacterial studies (in vitro) showed significant bacteriostatic action with a rate reaching 98% against *S. aureus* (Xu et al., 2019). Therefore, the finding presented in this paragraph displayed that such integrated nanosystems can be promising multifunctional platforms for achieving accurate detection and efficient eradication of *S. aureus* bloodstream infections.

Recently, multifunctional nanomedicines derived from a combination of chemo and photothermal treatments have garnered considerable interest due to their cooperatively enhanced bactericidal activity (Huo et al., 2021; Wei et al., 2020). In this regard, Ran et al. (2017) presented an HAase‐triggered photothermal nanoplatform for the eradication of bacteria based on AgNPs and graphene oxide (GO). First, The AgNPs were coated with HA to form nanocomposites with a size of 30 nm; subsequently, these NPs were integrated with GO to form GO‐HA‐AgNPs. Due to the presence of the HA layer, the nanocomposites exhibited low toxicity to host cells. Furthermore, the release behavior of HAase‐responsive AgNPs enabled AgNPs to be protected by the HA layer without affecting host cells and offer controlled release in the presence of bacteria. After HA was degraded, the sheetlike GO could accumulate on the surface of bacteria via physical interactions, and upon illumination of NIR light, the GO‐based sheet locally raised the temperature, resulting in significant bacteriostatic and bactericidal actions thus resulting in synergistic therapy. This hypothesis was confirmed by performing both in vitro and in vivo antibacterial studies, which showed that GO‐HA‐AgNPs had greater antibacterial activity compared to AgNPs (Ran et al., 2017). Two years later, another HAase‐responsive nanocomposite, AA@Ru@HA‐MoS2, with a synergistic chemo‐photothermal property was reported by Liu et al. (2019) to treat bacterial infections. In this study, HA‐coated mesoporous ruthenium nanoparticles (MRNs) were used as nanocarriers, ascorbic acid was loaded as a prodrug, and then ciprofloxacin‐molybdenum disulfide was integrated as a catalyst at the outer surface. The formulated nanocomposites were pompon‐like with a diameter of 206 nm. When AA@Ru@HA‐MoS2 reached the infection site, B‐HAase degraded the HA layer, releasing ascorbic acid and subsequently generating hydroxyl radicals in situ through molybdenum disulfide catalysis. Additionally, by exploiting the excellent photothermal properties of MRNs, a combination of chemo‐ and photo‐thermal antibacterial therapy could be accomplished. A set of in vitro antibacterial studies on two bacterial models, namely *S. aureus* and *P. aeruginosa*, indicated that AA@Ru@HA‐MoS2 not only had bactericidal activity, but also significant antibiofilm activity. The same finding was also obtained in the in vivo antibacterial experiment. Furthermore, the mouse infection model showed faster wound healing activity and excellent short and long‐term biosafety profiles. Overall, these studies indicate that HA‐coated antibacterial nanocarriers could further enhance the synergy of chemo‐photothermal treatments. Such functionalization represents a robust strategy for designing enzyme‐responsive multifunctional nanomedicines to improve targeted antibacterial delivery.

In summary, as HA is made up of enzyme‐degradable disaccharide units, it is considered an ideal candidate for the fabrication of nanocarriers with an HAase‐responsive crown. These HAase‐responsive NPs hold great potential for more precise detection and more effective therapy of bacterial infections.

### 4.3 Other hyaluronic acid‐coated nanocarriers

In addition to the aforementioned rationales for coating various antibacterial nanocarriers with HA, HA can also be used as a coating material to reduce the toxicity of metallic NPs as well as an antiadhesive agent to prevent adsorption of biomolecules on the surface of nanocarriers (Abdel‐Mohsen et al., 2013; Ivanova et al., 2018).

The application of colloidal Ag‐based materials has been rising owing to their potent antimicrobial action. Therefore, AgNPs have been used in several areas, including manufacturing cosmetics, medical devices, and pharmaceutical products (Knetsch & Koole, 2011; X.‐F. Zhang, Liu, et al., 2016). However, due to its toxicity, these applications are limited. Silver NPs have been widely studied to reach biocompatible and environmentally friendly products by capping AgNPs with various bioactive polymers (Mohamed Fahmy et al., 2019). Thus, coating AgNPs with HA could be a promising strategy for biomedical applications. In this regard, Abdel‐Mohsen et al. (2013) designed and prepared AgNPs‐capped with HA fibers (HA‐AgNPs) as a potential wound dressing material. Silver NPs with diameters of 10 and 40 nm were prepared, then coated with HA fibers. In this study, HA served as a capping agent for Ag^+^ as well as stabilizing agent during and after the synthesis of AgNPs. Several experiments were conducted to investigate the characterization, biocompatibility and cell viability, thermal profiles, and antibacterial activity of HA‐AgNPs. Using mouse fibroblast 3T3 cells, the MTT assay indicated that HA, HA fibers, and HA‐AgNPs were nontoxic to the cell line. Moreover, HA‐AgNPs showed potent in vitro antibacterial activity against *S. aureus* and *E. coli*. This work showed that modification of AgNPs with HA can minimize their cellular toxicity. Further in vivo evaluation is necessary; however, AgNPs embedded in HA fibers can be employed for a variety of biomedical applications, most notably wound healing and dressing (Abdel‐Mohsen et al., 2013).

Hyaluronic acid has been widely employed for surface engineering of nanomaterials to enhance their stability and functionalities for biomedical applications. One of the primary obstacles that face the optimized nanoformulations when administered in vivo is the biofouling effect. Biofouling is defined as nonspecific interactions between biomolecules and the engineered nanocarriers leading to in situ changes in their pharmacokinetics, pharmacodynamics, and toxicity. Among the polysaccharides studied as anti‐biofouling agents, HA is the most employed polysaccharide. Its antifouling actions appeared to be promising in terms of inhibiting nonspecific protein adsorption and enhancing nanocarrier stability in complex serum‐containing media (Lemarchand et al., 2004; Li et al., 2017; Mosaiab et al., 2019). Ivanova et al. (2018) accomplished this goal by coating bioinert polymeric NPs with several bilayers of biocompatible and antifouling HA and antibacterial aminocellulose. After five cycles of layer‐by‐layer coating technique, polyelectrolyte‐coated NPs (HA‐AC NPs) with a size below 50 nm were formed. The surface charge of the nanosystems is governed by its outermost layer. All coated NPs were shown to be biosafe when tested on human fibroblast cells. A series of in vitro antibacterial studies against *E. coli and S. aureus* were conducted. The results indicated that the coated NPs were capable of eradicating planktonic bacteria and inhibiting biofilm formation. For an exposure period of less than 1 h, the NPs with aminocellulose as the outermost layer demonstrated high antibacterial efficacy against gram‐positive and gram‐negative bacteria. On the other hand, when HA was used as the outermost layer of NPs, a significant increase in antibiofilm activity was found against tested bacteria (including *E. coli*). Although the exact antibiofilm mechanism of HA‐terminated NPs is not fully understood; it has been justified by the contribution of the HA layer (as anti‐biofouling) to the improved colloidal stability of the nanocarrier in tryptic soy growth media and consequently enhanced efficacy of NPs toward gram‐negative strains. This work demonstrated the anti‐biofouling property of HA in protecting nanocarriers from nonspecific interactions with serum proteins (Ivanova et al., 2018). However, further experiments (in vivo*)* should be pursued to study the potential of these NPs for treatment of infectious diseases.

Overall, surface functionalization with HA endows nanoparticles with bacterial targeting ability, enhances the colloidal stability and biosafety, protects the NPs from biofouling effects, and even serves as a gatekeeper to control the drug release. Table 2 summarizes all HA‐coated nanocarriers that have been used to date for antibacterial delivery. The table provides comprehensive information such as the size of the nanosystem, bacteria species, antibacterial agents(s), release triggering strategy, mode of therapy, and mode of study.

## 5 HYALURONIC ACID‐DRUG CONJUGATES

Hyaluronic acid is a large biocompatible hydrophilic polymer of repeating sugar units that can be covalently attached to various drugs (Arora et al., 2016; Prajapati & Maheriya, 2019). In 1991, the concept of drug conjugation to HA was introduced for the first time (Drobnik, 1991). In antibacterial delivery, direct conjugation of HA to various drugs could yield novel nanosystems with promising antibacterial effects (Arshad, Tabish, Kiani, et al., 2021; Lu et al., 2020; Qiu et al., 2019). Such straightforward yet effective nanoformulations can be employed to enhance therapeutic efficacy against intracellular pathogens, as many inflammatory cells overexpress HA‐targeted receptors (CD44). Furthermore, the covalent bonds between HA and the conjugated antibiotics can be tailored so they are not easily broken in the blood, but they are disrupted by hydrolysis and/or enzymatic degradation once they reach the target, releasing the drug. Thus, providing merits in terms of prolonging circulation time, increasing drug stability and solubility, and controlling release behavior (S. N. Kłodzińska & Nielsen, 2021).

**FIGURE 12:**
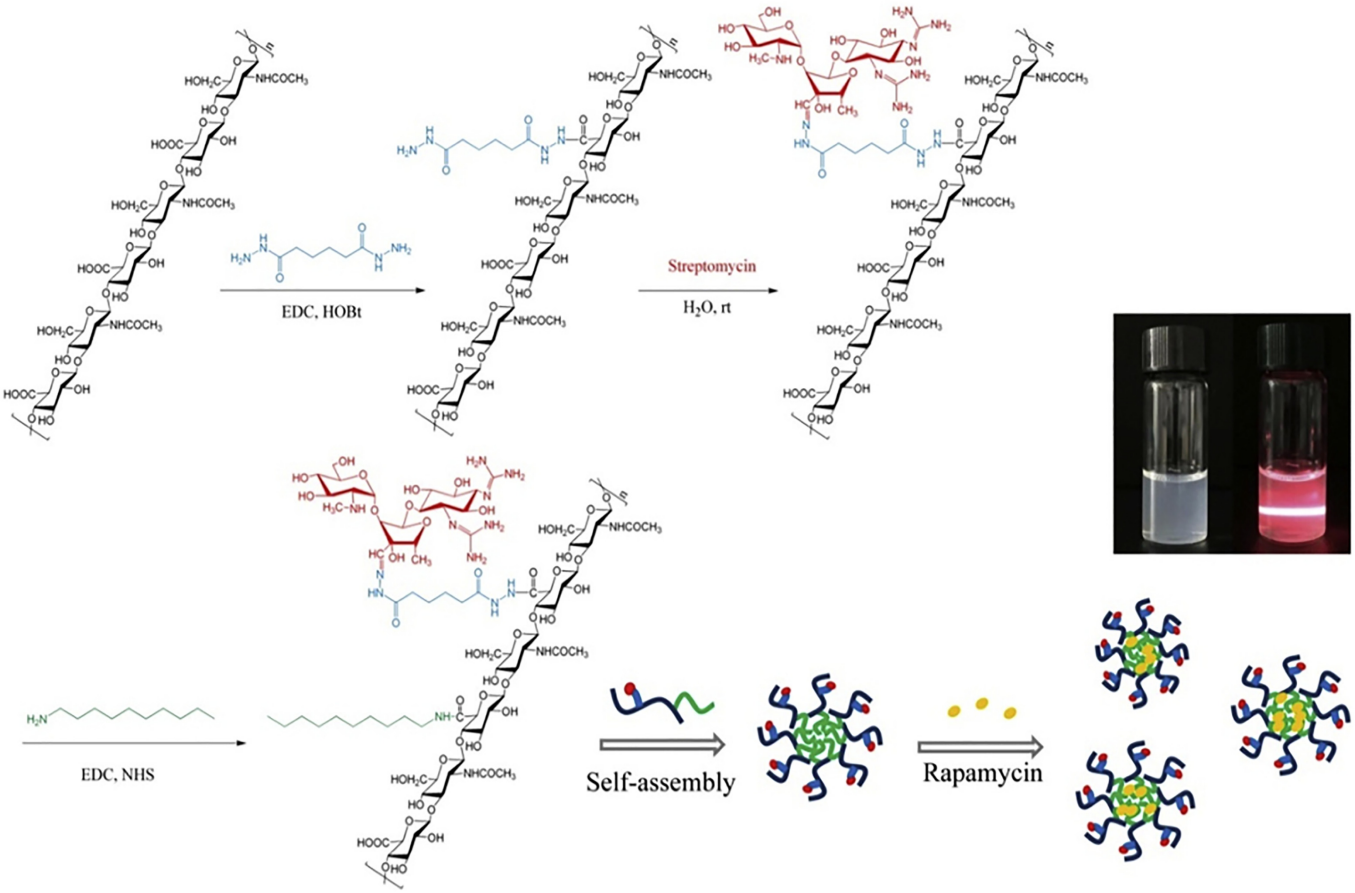
Schematic illustration of rapamycin‐loaded HA‐based micelles (Qiu et al., 2019)

Instead of conjugating macromolecular hydrophobic polymers, small lipophilic drug molecules can be grafted onto the N‐acetyl, hydroxyl, or carboxyl groups of HA. The synthesized HA‐drug conjugates can be self‐assembled into nanomicelles; thus, the conjugated drugs can be safely delivered to their destination (B. Chen, Miller, & Dhal, 2014). Even though antibiotics are powerful agents against pathogenic bacteria, more than 70% of them are ineffective against intracellular pathogens (Butler & Cooper, 2011). Accordingly, to combat these threatening infections, there is a dire need to invent novel antibacterial agents that can penetrate inside mammalian macrophages and efficiently eliminate these pathogens. In this regard, two studies by Jinyou Duan research group have been reported. In both studies, HA‐based micelles were constructed by conjugating lipophilic antibiotics with HA. Hyaluronic acid was used to maximize the uptake of the conjugated drug via a CD44‐mediated mechanism leading to improved antibacterial action (Lu et al., 2020; Qiu et al., 2019). In the former study, Qiu et al. (2019) designed and synthesized pH‐sensitive HA‐based micelles containing HA‐streptomycin conjugate and rapamycin, an autophagy activator (Figure 12). The novel self‐assembled compound was synthesized by grafting streptomycin and decylamine on HA (via acid‐labile bond) and subsequently loading with rapamycin via a spontaneous self‐assembly approach. The prepared HA‐based micelles had a hydrodynamic diameter of 179 nm with an EE% of 69.3%. At pH 5.5, the drug‐HA bond was cleaved, releasing streptomycin and the loaded rapamycin at the infection site. Moreover, using RAW 264.7 and HK‐2 cell lines, MTT assay validated the biocompatibility of blank and loaded micelles at concentrations below 400 μg/ml. The rapamycin‐loaded HA‐micelles demonstrated a high intracellular eradication rate against *Salmonella typhimurium* and *S. aureus*, even more than the equivalent mixture of streptomycin and rapamycin. As mentioned in literature, bare streptomycin cannot penetrate mammalian cells, thus exhibiting poor antibacterial action against intracellular microorganisms (Tulkens, 1991). Accordingly, conjugation of streptomycin with HA facilitates its uptake via CD44‐mediated endocytosis. In addition, the rapamycin could further potentiate the action of the system via activation of autophagy (Qiu et al., 2019). In the subsequent study, Lu et al. (2020) synthesized NO‐triggered micelles (HA‐NO‐LVF) by conjugating an HA chain and a lipophilic LVF followed by self‐assembly in aqueous conditions. It is known that when macrophages become infected, they generate a large amount of NO. Thus, exploiting endogenous NO to cleave HA‐conjugated nanocarriers increases the potential for not only responsive drug release but also for scavenging redundant NO radicals to reduce tissue injury. In that sense, the in vitro release study in the presence of NO demonstrated that the micelles were cleaved, releasing LVF in the pathogenic environment. Meanwhile, the NO‐responsive HA‐micelles retained hemocompatibility as well as biosafety profiles. Similar to their former study, HA‐NO‐LVF demonstrated significant antibacterial action against intracellular *S. aureus*, which could be due to CD44‐mediated endocytosis of HA‐NO‐LVF and subsequent on‐demand release of LVF, resulting in a higher eradication rate against intracellular bacteria. Moreover, in vivo evaluations acknowledged the ability of HA‐NO‐LVF to safely eliminate intracellular bacteria and reduce inflammatory mediators. In both studies, the assumption of CD44‐mediated uptake of nanosystems was validated via performing a competitive inhibition assay. The findings of the assay revealed that when the CD44 receptors were occupied by HA, the intracellular uptake of the conjugated HA‐micelles was dramatically reduced. Taken together, both studies proved the synergistic action of HA‐mediated uptake and the bactericidal action of the antibiotic in eradicating intracellular pathogens, hence lowering the required doses of used antibiotics (Lu et al., 2020; Qiu et al., 2019).

Self‐emulsifying drug delivery systems are isotropic mixtures of drugs, oil, surfactants, and co‐surfactants that form oil‐in‐water (O/W) nanoemulsions upon contact with water and mild agitation. The formed nanoemulsions are thermodynamically stable with nanosized droplets, high solubilization ability for hydrophobic drugs, and enhanced biocompatibility and pharmacokinetic profiles (Dalal et al., 2021; Kazi et al., 2019). Due to the overexpression of CD44 receptors on intestinal inflammatory cells, HA is an ideal choice for the preparation of HA‐based nanoemulsions targeting intestinal infections (Eriksson et al., 2003; Johnson & Ruffell, 2009). In this regard, Arshad, Tabish, Kiani, et al. (2021) successfully conjugated ciprofloxacin to the HA chain via an amidation reaction. The synthesized conjugate was delivered as a self‐nano emulsifying drug delivery system (HA‐CIP‐SNEDDS). The aim of this study was to improve ciprofloxacin mucopenetration, biopharmaceutical parameters, and hence intracellular antibacterial activity. The optimized nanoemulsion was found to be hemocompatible and biocompatible with a droplet size of 50 nm and maximum drug encapsulation efficiency of 85%. Moreover, at conditions mimicking the endosomal environment, HA‐CIP‐SNEDDS demonstrated superior and sustained release for 72 h in comparison to bare ciprofloxacin. As predicted, the fluorescence assay demonstrated that HA enhanced the internalization of ciprofloxacin by scavenger inflammatory cells, resulting in efficient targeting and eradication of *S. typhimurium*. Consequently, antibacterial studies showed that HA‐CIP‐SNEDDS had a strong bactericidal and antibiofilm activity against *S. typhimurium* compared to bare ciprofloxacin. When administered in vivo, HA‐CIP‐SNEDDS exhibited enhanced anti‐salmonella activity, confirming the in vitro results. Therefore, this study showed that HA‐CIP‐SNEDDS seems to be a promising antibacterial agent against *S. typhimurium* with a strong targeting ability (Arshad, Tabish, Kiani, et al., 2021).

These studies confirmed the potential use of HA as an antibacterial drug carrier to enhance targeted delivery and activity of conjugated antibiotics. This approach can significantly enhance antibiotic concentrations at infection sites, as well as cellular uptake and recognition of antibiotics, resulting in highly effective antibiotic targeted therapy.

## 6 CONCLUSION AND FUTURE PERSPECTIVES

Polymer‐based nanoantibiotics have been thoroughly explored to improve the efficacy of conventional antibiotics in various biomedical applications. Among all polymers used, HA has garnered considerable attention in the development of nanocarriers that can enhance the delivery of antibacterial agents to infection sites. In this review, we have highlighted and critically analyzed all reported nanosystems in literature that incorporate HA using different strategies for antibacterial applications such as treating, preventing, and/or diagnosing bacterial infections. After an extensive analytical search of various scientific databases, we have systematically categorized these HA‐NPs according to their approach for application into HA‐based nanocarriers, HA‐capped NPs, and drug‐conjugated HA‐NPs. Therefore, HA and its conjugates are considered promising candidates for developing targeted nanocarriers of therapeutic or imaging agents for bacterial infections.

**FIGURE 13:**
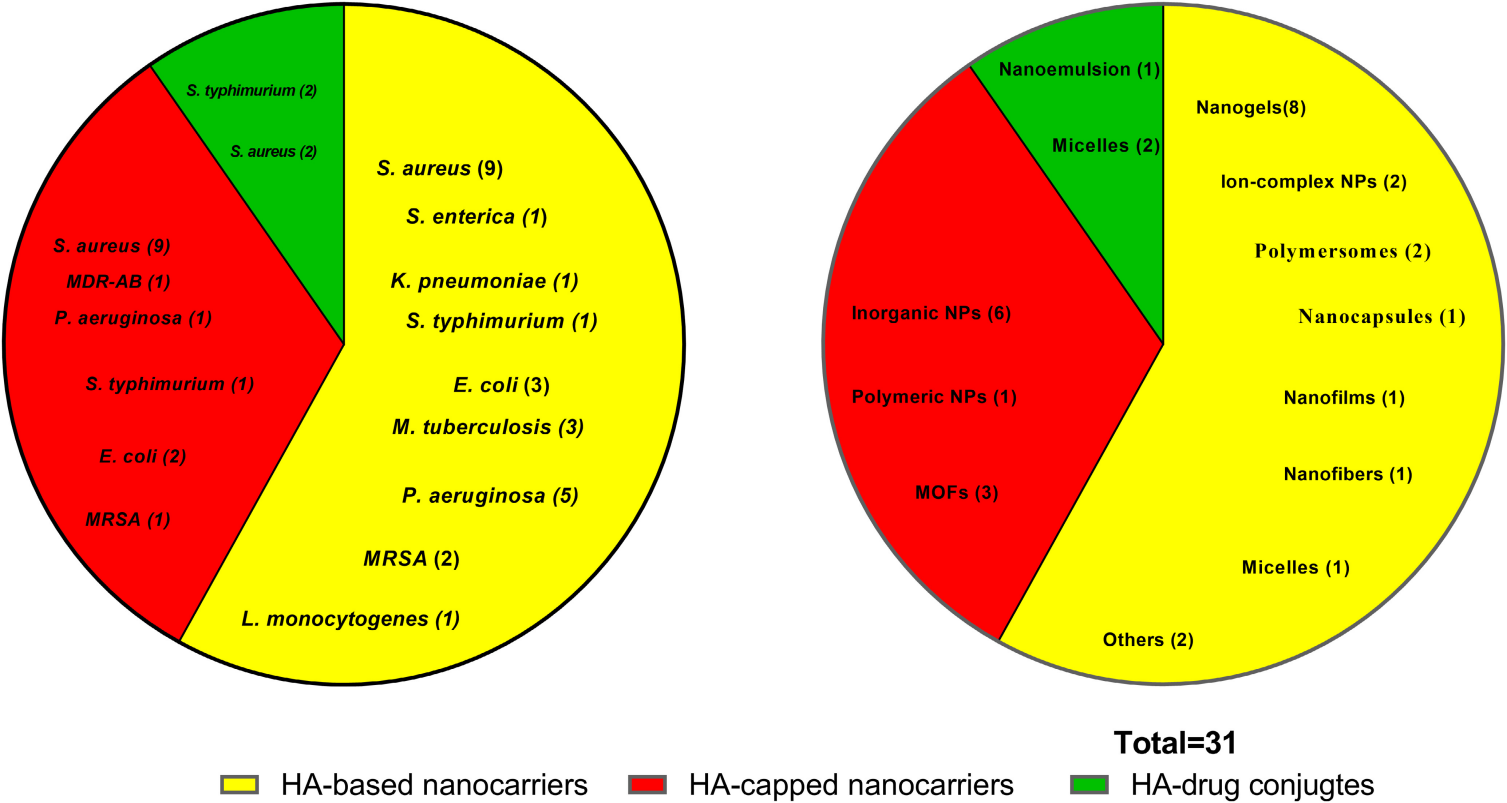
Hyaluronic acid‐based nanoparticles (right) that have been developed for the detection and/or eradication of various bacteria (left) over the last two decades

Based on the reported studies to date, we quantitatively analyzed and compared the various types of HA‐based nanocarriers that have been employed for improving antibacterial properties (Figure 13). It is apparent that HA‐based nanocarriers have been formulated and evaluated for their antibacterial potential, more than the other two strategies; and that the antibacterial activity of these nanocarriers has been tested against *S. aureus* and *E. coli*, more than other bacteria. This will aid future researchers in identifying the best strategy for preparing HA‐NPs as well as the infectious microorganisms on which to focus.

Sites of bacterial infection have hallmarks not present in uninfected tissues; they often include cells overexpressing CD44 receptors and have unique microenvironments such as acidic pH and high levels of specific enzymes (including B‐HAase). Therefore, HA‐NPs can enhance antibacterial delivery to eradicate intracellular infections such as TB, typhoid, brucellosis, and others. Moreover, the polymeric nature of HA enables prolonged circulation of loaded (or conjugated) antibiotic(s) in the bloodstream, resulting in sustained release behavior. After reaching the targeted area, initial burst release of antibacterial agents and activation of their functions at the infection site may occur in response to B‐HAase, thus enabling on‐demand release of the loaded drug(s). In addition, surface functionalization using HA could increase the biocompatibility, intracellular uptake, and colloidal stability of inorganic nanoparticles. Therefore, this strategy has a bright future in advancing the field of inorganic nanomedicines drug delivery to treat and diagnose bacterial infections. On the other hand, HA‐drug conjugates are considered as prodrugs synthesized by covalently attaching small antimicrobial drugs to HA. These linkages are not easily broken in the blood; however, they are breakable through acidic or enzymatic hydrolysis after reaching the target and releasing the drug. Therefore, such conjugates can serve as a targeting ligand for CD44 receptors on the surface of macrophages, allowing antibiotics to be safely delivered to eliminate intracellular infections.

While considerable efforts have been invested in proving the enhanced potential of HA‐NPs for bacterial eradication and detection, these nanosystems are still in their laboratory research stages, and numerous further studies are necessary to obtain regulatory approval. The production, synthetic modification, and precise characterization and optimization of HA‐NPs, as well as their biosafety and in vivo pharmacokinetics, should be carried out accurately according to clinical requirements. Moreover, more efforts are required to overcome obstacles associated with the process of scaling up these formulations into cost‐effective products. Additional investigations, including in silico molecular dynamics and in vitro binding affinity calculations (using microscale thermophoresis), are required to fully understand the interactions between these nanocarriers and the targeted biomolecules. Furthermore, the in silico studies can help to determine the stability of the synthesized HA‐NPs using virtual conditions that mimic experimental conditions.

Coating the surface of nanocarriers with HA is an effective strategy for enhancing antibacterial delivery as it provides anti‐adhesion properties, colloidal stability, and selective targeting ability. In addition, the secretion of HAase by various bacteria can be exploited by creating multifunctional HA‐coated NPs that can detect and treat HAase‐secreting bacteria. These multifunctional nanocarriers have the potential to revolutionize the diagnosis and treatment of HAase‐related infections. As a result, more research efforts should be focused on the surface modification of nanocarriers with HA. Furthermore, targeting bacterial virulence factors via mimicking cell signaling pathways may enhance antibacterial efficacy of loaded drugs and minimize their adverse effects and thereby, contribute to tackling the pathogenesis of infection. Therefore, by synthesis of modified HA‐NPs targeting B‐HAase (as a virulent factor), it is possible to inhibit bacterial pathogenicity. However, up to the date of this review, no research has been done in order to develop HA‐NPs with this biomimetic action, leaving huge research areas to be explored. Generally, formulating HA‐drug conjugates is the simplest form in terms of preparation and validation; however, in antibacterial delivery, only three studies have been reported. Therefore, it is expected that the investigations particularly in the area of intracellular antibacterial delivery will be expanded, since HA can facilitate the internalization of conjugated antibiotics via CD44 receptors and provide “on‐demand” release at the infection site. Based on the potential shown in this review, we envisage an explosion in the use of HA for transforming diagnosis and treatment of bacterial infections. Hyaluronic acid as a key excipient in nanomedicines has the potential to save lives and improve quality of life.

## CONFLICT OF INTEREST

The authors have declared no conflicts of interest for this article.

## AUTHOR CONTRIBUTIONS

**Mahir Mohammed:** Conceptualization (lead); data curation (lead); formal analysis (lead); investigation (lead); methodology (lead); software (lead); validation (equal); visualization (lead); writing – original draft (lead); writing – review and editing (lead). **Nikita Devnarain:** Conceptualization (equal); data curation (equal); formal analysis (equal); methodology (equal); writing – review and editing (equal). **Eman Elhassan:** Data curation (supporting); methodology (supporting); writing – original draft (supporting); writing – review and editing (supporting). **Thirumala Govender:** Conceptualization (equal); funding acquisition (equal); supervision (equal); validation (equal); writing – review and editing (equal).

## ETHICS STATEMENT

*Animal studies*: No animal or human studies were carried out by the authors of this article.

## RELATED WIREs ARTICLES

Intrinsic stimuli‐responsive nanocarriers for smart drug delivery of antibacterial agents: An in‐depth review of the last two decades

Surface modification of nano‐drug delivery systems for enhancing antibiotic delivery and activity

Inflammation‐modulating nanoparticles for pneumonia therapy

## Data Availability

Data sharing is not applicable to this article as no new data were created or analyzed in this study.
